# Characterisation of an allosteric site in PLCγ enzymes and implications for development of their specific inhibitors

**DOI:** 10.1042/BCJ20253358

**Published:** 2025-10-16

**Authors:** Tom D. Bunney, Hunter G. Nyvall, Calum Macrae, Damjan Lalović, Ashley Gregory, Kyle I. P. Le Huray, Nikita Harvey, Nikos Pinotsis, Antreas C. Kalli, Christopher A. Waudby, John E. Burke, Matilda Katan

**Affiliations:** 1 Institute of Structural and Molecular Biology, Division of Biosciences, University College London, London, WC1E 6BT, UK; 2 Department of Biochemistry and Microbiology, University of Victoria, Victoria, BC, Canada; 3 Leeds Institute of Cardiovascular and Metabolic Medicine, School of Medicine, University of Leeds, Leeds, LS2 9JT, UK; 4 UCL School of Pharmacy, Brunswick Square, London, WC1E 6BT, UK; 5 Institute of Structural and Molecular Biology, Birkbeck College, London, WC1E 6BT, UK; 6 Department of Biochemistry and Microbiology, University of British Columbia, Vancouver, BC, Canada; 7 University of Victoria, Genome BC Proteomics Centre, Victoria, BC, Canada

**Keywords:** allosteric regulation, inhibition, mutation, phospholipases, structural analysis

## Abstract

Phospholipase C gamma (PLCγ) enzymes are key components of intracellular signal transduction processes and are involved in disease development, including immune dysregulation, specific cancer types and neurodegeneration. Although recognised as important targets for intervention, validated pharmacological tools are lacking. Here, we demonstrate that inhibitory nucleotides bind directly to an allosteric site at the interface between the PLC-core and regulatory-array unique for PLCγ, underlying their specificity for the PLCγ family. This binding site overlaps with the PLCγ autoinhibitory interface, suggesting that the inhibitory impact of nucleotides involves stabilisation of autoinhibition. We have also analysed disease-linked variants of PLCγ1 and PLCγ2 to show that multiple mechanisms could underpin their gain-of-function phenotype. While the sensitivity of these variants to physiological nucleotide inhibition is reduced, we identified artificial nucleotide compounds that can inhibit such variants not only *in vitro* but also in cell-based assays. Therefore, our findings suggest a route for development of isozyme specific PLCγ inhibitors allowing further studies of their roles in health and disease.

## Introduction

Phospholipase C gamma (PLCγ) enzymes have long been recognized as key components in intracellular signal transmission, linking activation of several types of cell-surface receptors, such as receptors tyrosine kinases (RTKs) and immune cell receptors, to downstream processes triggered by the PLC-generated second messengers, diacylglycerol (DAG) and inositol(1,4,5)trisphosphate (IP_3_) [1–3]. During the last ten years, their roles in disease development have also become apparent. Notably, extensive genetic studies have revealed a PLCγ subnetwork as an important regulator of cell functions that can be subverted in various diseases. Furthermore, for many of these diseases, current treatments are ineffective and targeting PLC enzymes directly could address this unmet need.

Mutated variants of the two PLCγ enzymes, PLCγ1 and PLCγ2, have been linked to specific cancers (including T-cell lymphoma and angiosarcoma), resistance to cancer treatment (e.g. CCL resistance to ibrutinib), complex, dominantly inherited immune disorders (originally designated as PLAID and APLAID) and inflammation as well as with protection in Alzheimer’s and related neurodegenerative diseases; the major, comprehensive discoveries are described in [4–9]. There are also examples of aberrant signalling in a disease context that presumably involves PLCγ enzymes in their wildtype (WT) form [2].

Crucial to functional studies and studies of disease mechanisms are validated pharmacological reagents directed to the biological functions of a protein of interest. Furthermore, such compounds serve as candidates for drug development. The lack of such reagents targeting PLCγ enzymes or other PLC families is a well-recognised bottleneck in understanding their contribution to physiological responses, disease development and generation of new therapeutic modalities. Substantial literature has revealed that many compounds frequently used as PLC inhibitors (notably U-73122) do not directly target PLC enzymes [1]. Until relatively recently, the main limitations in the development of validated, direct PLC inhibitors or activators were related to a lack of suitable high-throughput screening (HTS), difficulties of generating chemical probes based on PI(4,5)P_2_ substrate and, importantly, a lack of motivation based on insufficient evidence linking changes in PLC function with disease development. In the last ten years, significant progress has been made in substantiating the involvement of PLCγ enzymes in a range of disease conditions described above as well as in the development of new PLC assays suitable for HTS [1,2]. One of the technologies that has been used to develop new assays for sensitive measurements of PLC products generated in cells, specifically the inositol-1-P accumulated following the conversion from IP_3_ in the presence of LiCl, is based on homogeneous time-resolved fluorescence (HTRF). This assay has also been adapted for measurements of PLC activity *in vitro*, using detergent-mixed micelles or lipid vesicles [10–12]. Other, now frequently used PLC assays *in vitro* are based on fluorogenic substrate mimetics that can be cleaved either in solution (e.g. aldol-518 myo-inositol-1-phosphate) or incorporated in lipid vesicles [PI(4,5)P_2_ analogue compound, XY-69] [11–13]. Despite this progress, there are currently only a few reports covering relatively small screens that mainly demonstrated feasibility of the assays for the HTS format [13–15].

An important aspect when considering pharmacological modulators of the PLCγ enzyme activity that can be further developed and used as drugs is a requirement for selectivity among 13 classical human PLC isozymes from 6 distinct families [1]. It is well documented that different PLC isozymes have diverse and sometimes opposing roles in the same disease and, importantly, that they have numerous and essential physiological functions [1,2]. In general, a common, targetable site in signal transduction proteins doesn’t preclude generation of selective inhibitors. Comprehensive structural insights into isozymes from different PLC families revealed a strongly conserved binding site for the substrate headgroup and Ca^2+^ [1,3]. However, a number of amino acid residues in the vicinity, including those implicated in interactions with the substrate lipid chains or cellular membrane, vary among different isoforms and could provide selectivity for PLCγ1 or PLCγ2 [3]. So far, supporting evidence for selective targeting of the PLC active site is lacking. Another possible route to achieve selectivity is by targeting known sites involved in regulatory protein–protein interactions. One such example is targeting the CD95 receptor that interacts directly with PLCγ1 in T cells [16]. A known drug molecule (ritonavir) and a synthetic peptidomimetic of the key region in the receptor (compound DB550) disrupt the CD95–PLCγ1 interaction, selectively inhibiting the pathway and alleviating clinical symptoms in a disease mouse model [17,18]. Other possibilities for selective targeting are based on PLC 3D structures and related functional studies that demonstrated that the activation state of different PLCs is stringently regulated *via* diverse autoinhibitory interactions that are released by changes in unique allosteric networks. These observations suggest that the selectivity can be achieved by targeting autoinhibitory and allosteric sites. While targeting autoinhibitory interfaces remains largely unexplored, there are reports of allosteric inhibition of PLCγ by nucleotides [13,19] with a recent study suggesting selectivity for inhibition by ATP [13]. However, the site of nucleotide binding and its suitability for guiding further drug discovery have not been explored.

In the present study, we characterise an allosteric binding site involved in PLCγ inhibition by ADP and ATP, providing insights into molecular mechanisms of selective inhibition of this PLC family. We further assess possible physiological implications of these findings and feasibility of targeting the allosteric site in PLCγ enzymes and their disease-linked variants by synthetic nucleotide analogues.

## Results

### Inhibition of PLCγ1 by ADP and ATP, direct binding and mapping of the binding site

**Figure 1:**
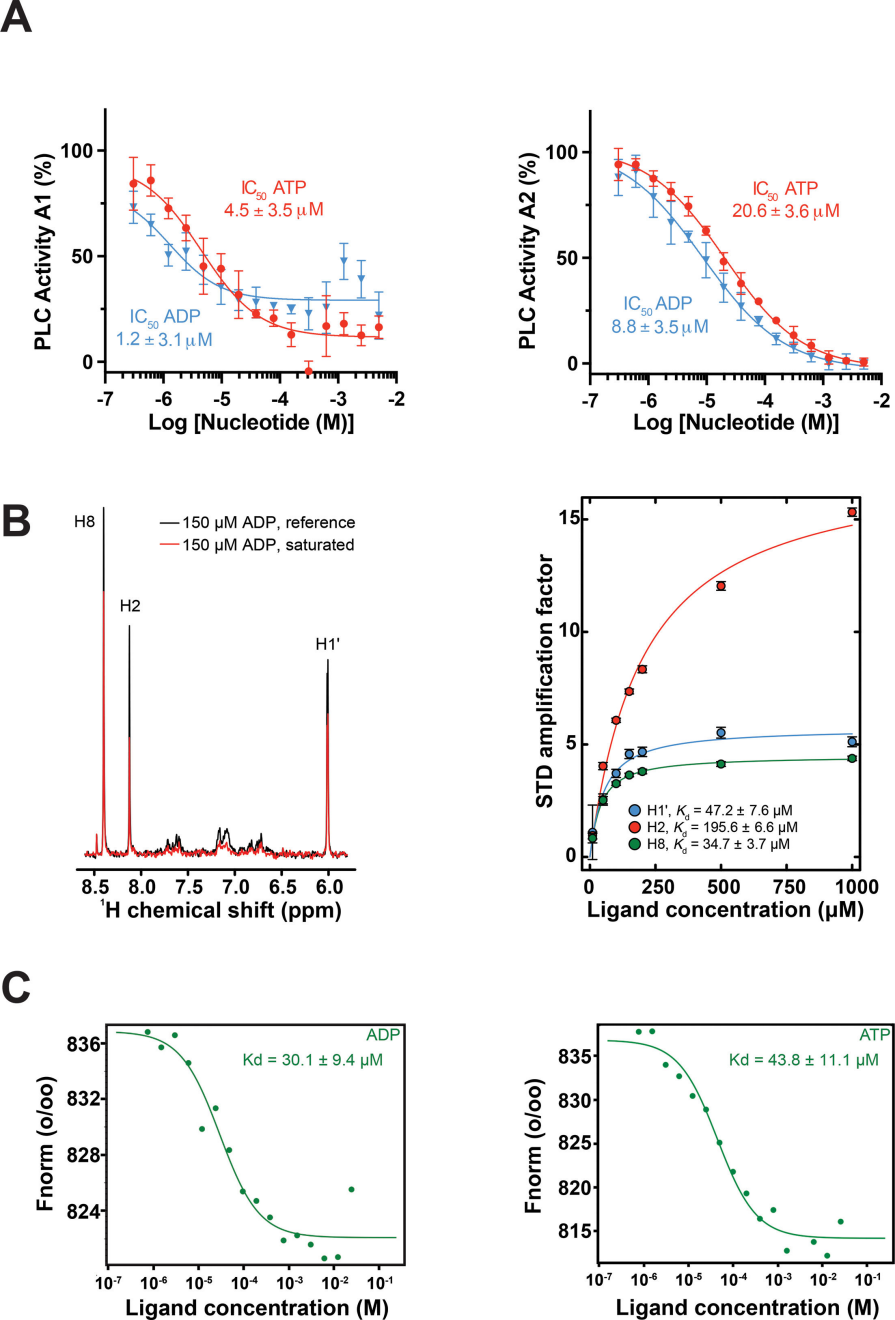
Adenine nucleotides inhibit and directly bind PLCγ1. (**A**) Inhibition of PLCγ1^wt^ by ATP and ADP using the XY-69 substrate embedded in liposomes with the lipid composition mimicking the plasma membrane (assay 1, **A1**) (left panel) and soluble substrate aldol-518 myo-inositol-1-phosphate (assay 2, **A2**) (right panel). IC_50_ values for each assay are presented. Data are from nine replicates and represents the mean and SD. (**B**) STD-NMR of ADP with PLCγ1^wt^ showing reference (black) and 2 sec saturation (red) spectra (left panel). STD amplification factors are presented for three proton resonances as a function of ADP concentration together with the fitted dissociation constants (right panel). The error bars represent the standard deviation (**C**) MST dose response curve of the interaction between ADP (left panel) or ATP (right panel) and PLCγ1^wt^. Data shown are representative for three independent MST measurements. The resulting dose response curves were fitted to a one-site binding model to extract *Kd* values (F_norm_ = normalized fluorescence).

In our initial studies, we found that both ATP and ADP inhibit PLC activity of PLCγ1^wt^ and further assessed both nucleotides in assays 1 and 2 (A1 and A2) where the substrate is either incorporated into liposomes (XY-96) (A1) or hydrolysed in solution (aldol-518 myo-inositol-1-phosphate) (A2) (Figure 1A). With respect to parameters that contribute to the overall values for PLC activity in these assays, it appears that only in assay 1 (A1) the exposure of membrane interaction surfaces and association with lipid structures have an important impact. As shown here, a previously described variant of the PLCγ1-core (deletion of residues 488–933), lacking key membrane interaction residues, (PLCγ1-core M) [11] (Supplementary Figure S1A), showed a reduction in PLC activity in A1 but had no considerable effect in A2 (Supplementary Figure S1B). ATP and ADP inhibited PLCγ1 in both assays with the IC_50_ values within the 1–20 μM range (Figure 1A). ADP was somewhat more potent (IC_50_ values about 2–4-fold lower); however, when using liposomes, stronger inhibition was observed for ATP at higher concentrations (higher than 15 μM). When comparing the two assays, inhibition by ATP and ADP was more pronounced when incorporating the PLC substrate into liposomes, with the IC_50_ value for ATP and ADP about four-fold and eight-fold lower, respectively. Nevertheless, our finding that the inhibition by these nucleotides can be readily detected in a simple assay presenting a substrate in solution (Figure 1A) suggests the nucleotide effect on multiple steps involved in efficient substrate hydrolysis.

To demonstrate that the inhibition of the PLCγ1^wt^ enzyme activity by the nucleotides results from their direct binding to the protein, we first used saturation transfer difference (STD) NMR measurements [20] with ADP as a ligand and observed transfer of saturation providing unambiguous evidence of binding (Figure 1B). Measurement and analysis of amplification factors for ADP signals as a function of concentration determined dissociation constants (*Kd*) between 30 and 50 μM for the H8 and anomeric H1′ resonances. The H2 resonance gave a higher value (~196 μM), likely an overestimate due to known experimental factors that affect STD measurements at fixed saturation times [21]. To further validate these findings, microscale thermophoresis (MST) was used, which confirmed *Kd* values in the range of 30–50 μM for ADP and ATP (Figure 1C).

**Figure 2:**
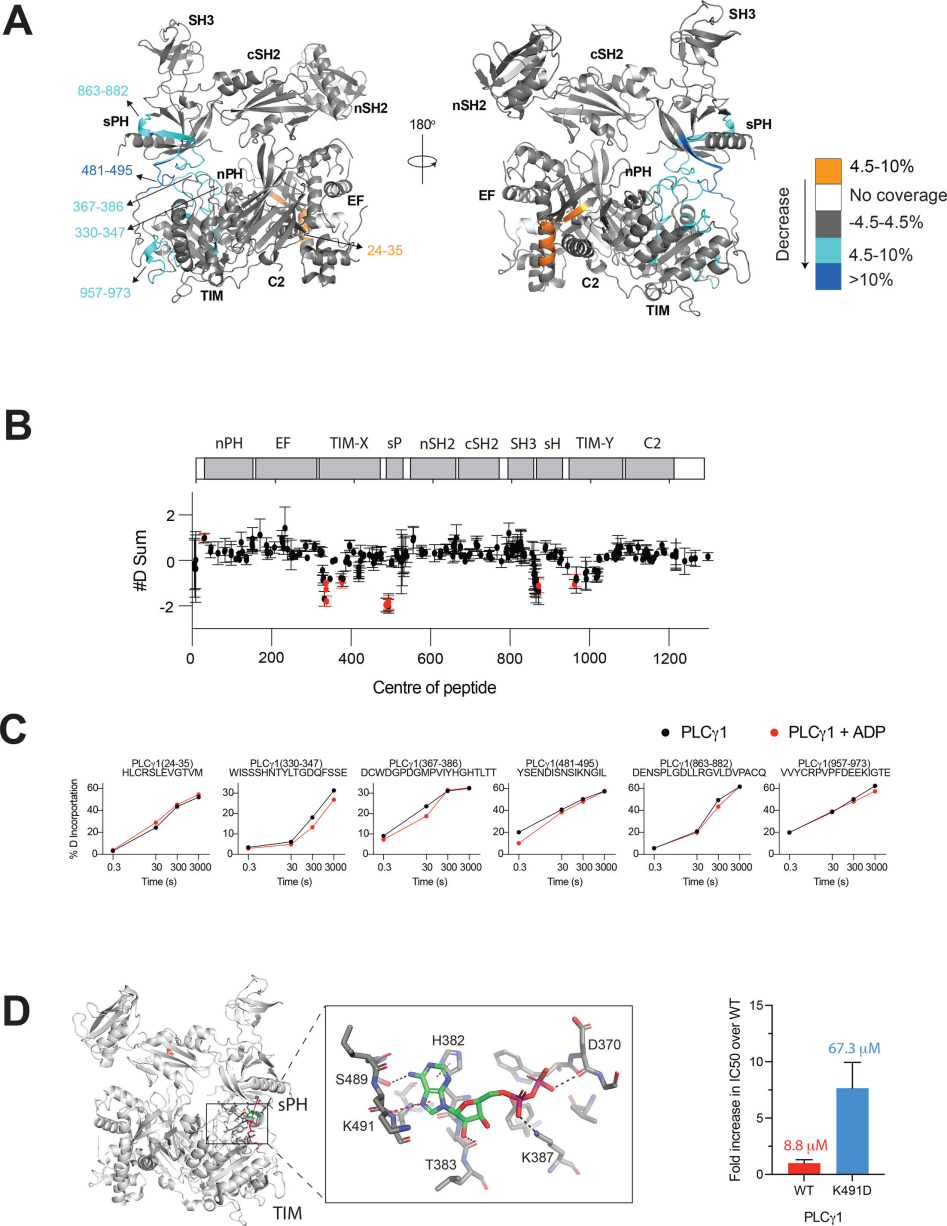
Nucleotide binding pocket is at the autoinhibitory interface between the TIM barrel and sPH. (**A**) HDX-MS analysis of the interaction between PLCγ1^wt^ and ADP. A Pymol generated representation of the structure of PLCγ1^wt^ showing the significant differences in deuterium incorporation when comparing apo PLCγ1^wt^ to PLCγ1^wt^ + 10 µM ADP, changes are colour coded according to the legend. (**B**) The sum of the #D difference upon addition of ADP shows the total difference in deuterium incorporation over the entire hydrogen-deuterium exchange time course, with each point indicating a single peptide (error shown as SD [*n* = 3]). Individual peptides with a significant difference between conditions (defined as greater than both a 4.5% and 0.45 Da difference in exchange at any time point, and a two-tailed, unpaired t-test of *P*<0.01) are coloured red. (**C**) A selection of peptides showing significant differential exchange between Apo PLCγ1^wt^ (black) and the complex of PLCγ1^wt^ and ADP (red) at any time point. Experiments are graphed as the mean percentage of deuterium incorporation at each time point with error shown as SD (*n* = 3). Most error bars are smaller than size of the point. The full H/D exchange MS data is available in the source data. (**D**) Docking of ADP onto the structure of PLCγ1^wt^ using the software Maestro. One docking outcome that locates the nucleotide at the TIM-sPH interface is shown (left panel). A close up of the docked ADP with interacting amino acid residue side chains highlighted (middle panel). Histogram illustrating the effect of mutating an interacting amino acid, K491, on the efficacy of inhibition on PLCγ1^wt^ by ADP (right panel). The PLC specific activity of the K491D variant is not affected (WT: 18606 ± 685 RFU/min/mg and K491D: 18974 ± 796 RFU/min/mg).

Following our initial, unsuccessful efforts to determine the nucleotide binding site using X-ray crystallography, we applied hydrogen deuterium exchange mass spectrometry (HDX-MS) that can capture protein states and interactions in solution [22]. We observed significant differences in deuterium incorporation (defined as >4.5%, > 0.45 Da and *P*<0.01 between conditions at any time point) when comparing PLCγ1 in the presence and absence of 10 µM ADP, defining an area with reduced exchange that could represent protection resulting from the ligand binding (Figure 2 and Supplemental source data). The area of protection, showing 4.5–10% (amino acids 330–347, 367–386, 863–882 and 957–973) or >10% (amino acids 481–495) difference in exchange, covers regions of the TIM barrel, sPH domain and their linker. Additionally, one small, separate area (amino acids 24–35 in the nPH domain) shows an increase in exchange (4.5–10% difference), probably resulting from allosteric conformational change that accompanies nucleotide binding.

Using molecular docking of ADP within the area corresponding to the reduced hydrogen deuterium exchange, we identified potential residues that could be involved in interaction with the ligand (Figure 2D, left panel). Further site-directed mutagenesis confirmed that one of the implicated residues tested, K491, was required for an efficient inhibition by ADP; the mutation (K491D) resulted in substantial reduction in the inhibition, without an effect on the PLC activity in the absence of ADP (Figure 2D, right panel). The lack of an effect on the PLC activity of this mutation, in a wider region known to incorporate activating mutations (e.g. S345F), makes indirect perturbations an unlikely cause of the observed reduced inhibition.

In addition to ADP, we performed similar docking of ATP and showed very good overlay of the two nucleotides (Supplementary Figure S2A). Furthermore, measurements of ADP and ATP binding using MST demonstrated a large reduction in nucleotide binding by the PLCγ1^K491D^ variant, compared with the WT shown in Figure 1C; estimated *Kd* values for ADP were ~1–2 mM and for ATP ~700 μM. Together with similar functional effects of the two nucleotides on PLCγ1, PLCγ2 and their variants, shown in the second result section, these data support that ADP and ATP have a common binding site.

The area in PLCγ1 highlighted as a likely nucleotide binding site, using HDX-MS and additional experimental approaches (Figure 2A–D; Supplementary Figure S2A), overlaps with one of the two autoinhibitory interfaces, an interface formed by interactions between the TIM barrel in the PLC-core and the sPH domain from the regulatory (γSA) region. However, the residues predicted to interact with nucleotides are distinct from the key residues that contribute to the autoinhibition at the TIM barrel/sPH domain interface [11]; based on this, the nucleotide binding is not expected to interrupt autoinhibition. Considering that ADP and ATP actually inhibit the PLC activity of PLCγ1 (Figure 1A), the ligand binding in this area is therefore likely to stabilise and enhance autoinhibition and, in the absence of stimulation/activators, further shift equilibria towards an inactive form. A strongly conserved PI(4,5)P_2_ headgroup/Ca^2+^ binding site is in a different, adjacent area of the TIM barrel [11] and the nucleotide binding is not predicted to directly compete with substrate binding. Consistent with this, kinetic analyses suggest a non-competitive nature of the nucleotide inhibition (Supplementary Figure S2B). However, as further considered in Discussion, we cannot exclude other allosteric effects not related to autoinhibition.

### Selectivity for PLCγ isozymes and the effect of disease-linked mutations on PLC activation and inhibition by ADP and ATP

**Figure 3:**
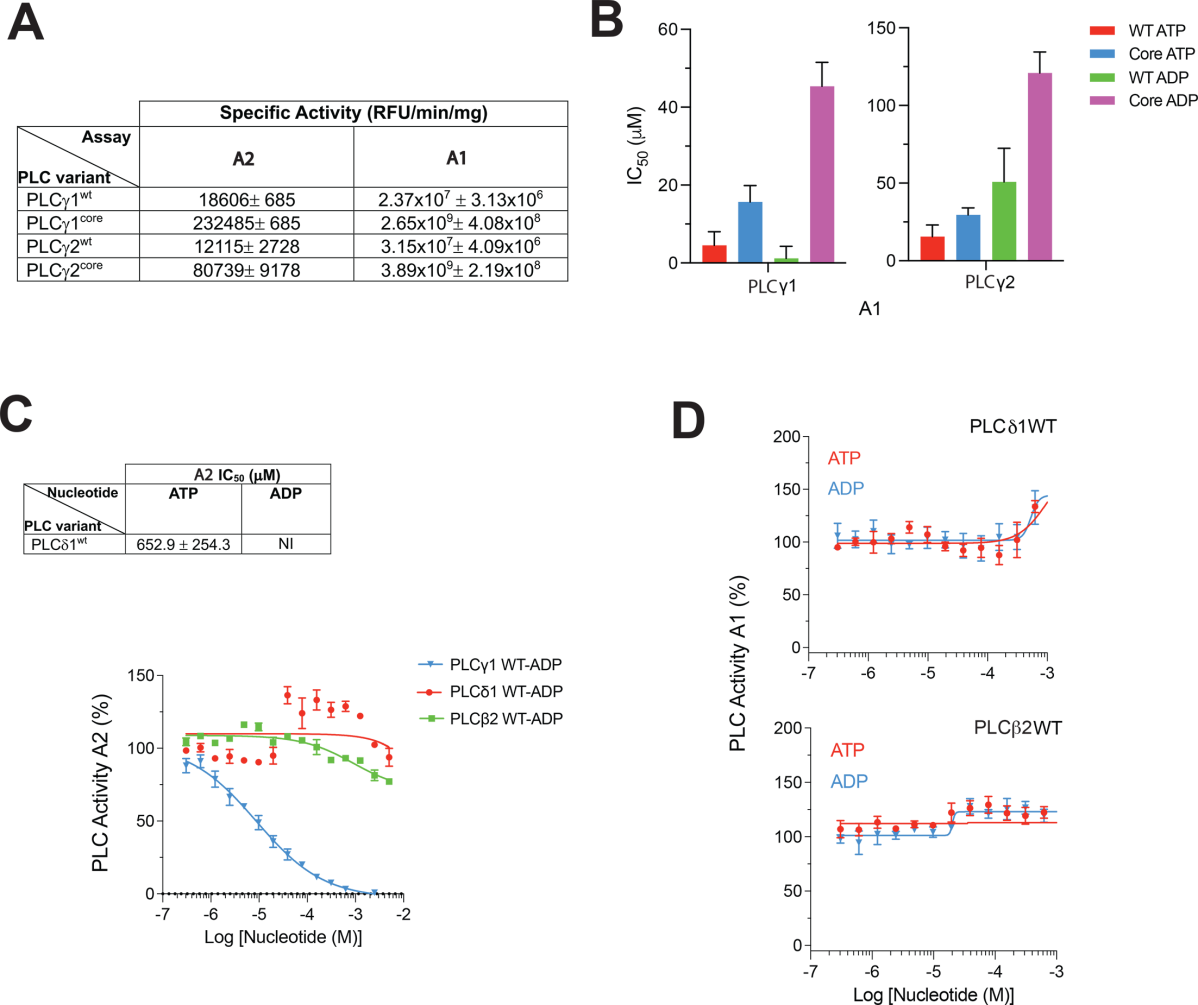
The nucleotide inhibition is specific for the PLCγ family with some differences between PLCγ1 and PLCγ2. (**A**) Table showing the specific activities of PLCγ1^wt^ and PLCγ2^wt^ full-length proteins and their corresponding core (regulatory SA removed) variants. (**B**) Histograms showing the IC_50_ values of ATP and ADP inhibition of PLCγ1^wt^ and PLCγ2^wt^ and their core variants in assay 1 (**A1**). (**C**) Table showing the IC_50_ values of ATP and ADP inhibition of PLCδ1 (top panel) and comparison of the inhibitory effect of ADP on 3 PLC isoforms (bottom panel) in assay 2 (**A2**). (**D**) The effect of ATP and ADP on the activity of PLCδ1 (top panel) and PLCβ2 (bottom panel) in assay 1 (**A1**). All experiments were performed in at least triplicate. The data shown represent the mean and SD.

Comparison of the PLCγ1 region implicated in the nucleotide binding with the corresponding region in PLCγ2 suggests only partial conservation (Supplementary Figure S3A). Because the putative nucleotide binding site includes residues from the TIM barrel and the sPH domain, when comparing PLCγ1 and PLCγ2 isozymes, we analysed the WT proteins as well as variants (PLCγ^core^) where the regulatory region containing sPH domain has been removed. As previously described [11,12], the PLC activity of the core variants, assessed in two *in vitro* assays, was substantially enhanced, with the biggest differences observed in the assay using liposomes (Figure 3A). In this assay, inhibition of PLCγ2^wt^ by ATP and ADP was weaker compared with PLCγ1^wt^ (IC_50_ values about 1.5- to 40-fold higher); additionally, ATP was a stronger inhibitor (Figure 3B). Nevertheless, for both PLCγ isozymes, the inhibition by nucleotides was reduced (up to 40-fold) when the regulatory region was deleted in the PLCγ1^core^ and PLCγ2^core^ variants. These data show that although the nucleotide binding and inhibition somewhat vary between the isozymes, intact PLCγ proteins are required for their full inhibitory impact. This is consistent with the allosteric nucleotide binding site being formed within a pocket between the core and regulatory regions (Figure 2). Comparison of PLCγ1 and PLCγ2 and the inhibition by the two nucleotides revealing some differences (Figure 3A and B) is consistent with similar but not fully conserved binding sites.

Further comparison with the isozymes from other PLC families, PLCδ1 and PLCβ2, confirmed that the inhibition by nucleotides assessed by substrate hydrolysis in solution (Figure 3C) or substrate presented in lipid vesicles (Figure 3D) was selective for the PLCγ family, as previously suggested [13]. Except for small changes at very high concentration of nucleotides, the PLC activity of PLCδ1 and PLCβ2 was not affected in the presence of ATP or ADP. Although a more extensive panel of PLC isozymes would provide more comprehensive experimental support to this claim, our understanding of the nucleotide binding site (Figure 2) applied to structural insights covering members of most PLC families [1] is consistent with selectivity for PLCγ.

**Figure 4:**
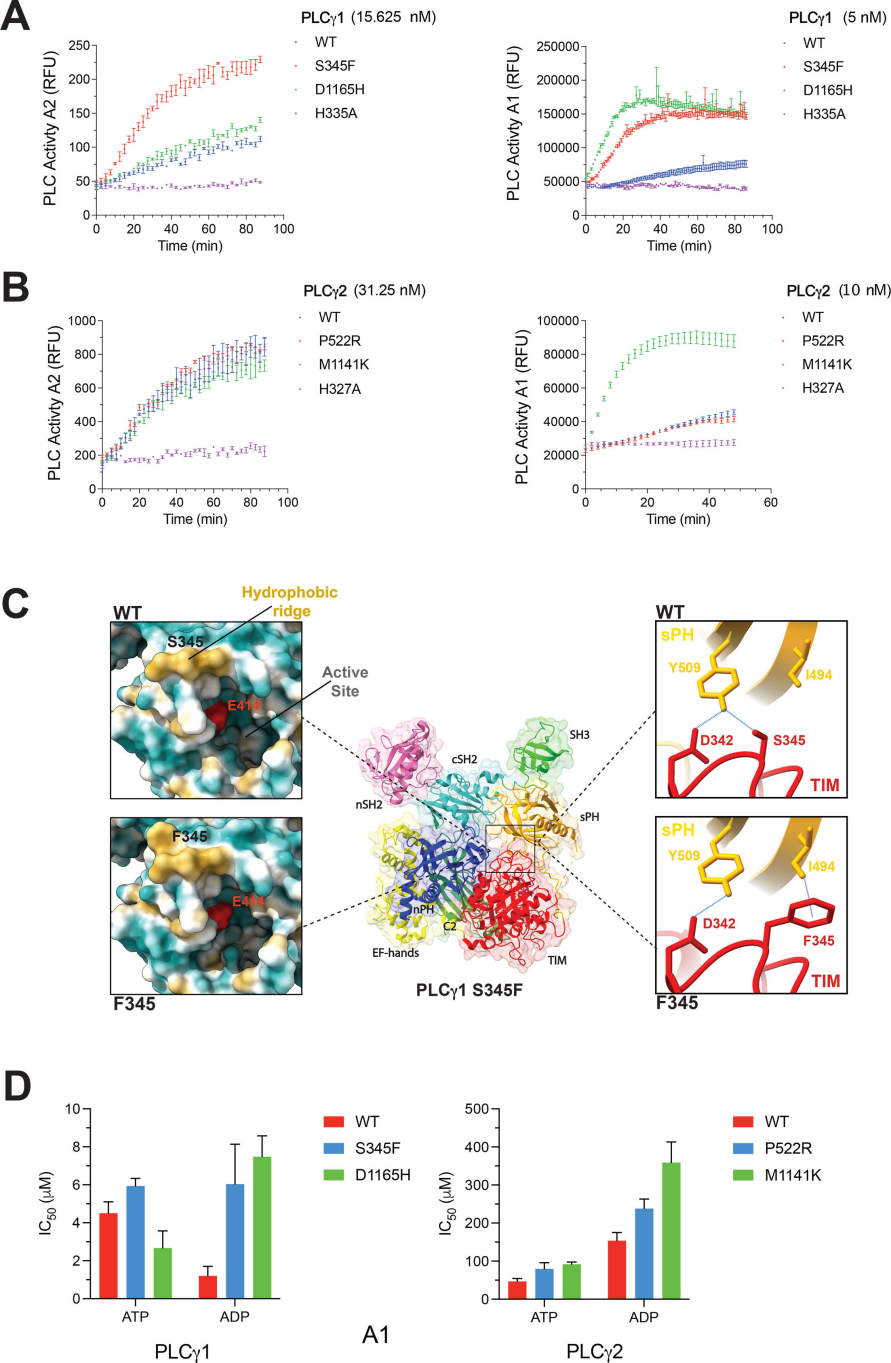
Activation mechanisms in PLCγ disease-linked variants vary, and the activation affects nucleotide inhibition. (**A**) Enzyme progress curves of PLCγ1 and its variants at a fixed enzyme concentration in assay 2 (**A2**) (left panel) and assay 1 (**A1**) (right panel). Data were generated in triplicate and the means and SD are shown. (**B**) Enzyme progress curves of PLCγ2 and its variants at a fixed enzyme concentration in assay 2 (**A2**) (left panel) and assay 1 (**A1**) (right panel). Data were generated in triplicate and the means and SD are shown. (**C**) Crystal structure of the PLCγ1^S345F^ variant (PDB ID: 9QB7; similar to the PLCγ1^wt^, PDB ID: 7Z3J, with the RMSD of 0.249 Å) (middle panel). An effect of the S345F mutation on a hydrophobic area (hydrophobic ridge) of the TIM barrel is shown as a surface representation with hydrophobic residues in yellow and charged residues in blue. Positions of the S345 and F345 as well as one of the active site residues (E414) are indicated (left panels). An effect of the mutation on interactions at the sPH/TIM barrel autoinhibitory interface, highlighting the S345, F345, D342 and I494 residues and their interactions (right panels). Analyses of the effects of the mutation are based on comparison of PLCγ1^wt^ and PLCγ1^S345F^ crystal structures (PDB 7Z3J and 9QB7, respectively). (**D**) Histograms showing the IC_50_ values of ATP and ADP inhibition of variants of PLCγ1 (left panel) and PLCγ2 (right panel) in assay 1 (**A1**). Each experiment was repeated at least three times and data show the mean IC_50_ and errors are SD.

Many disease-linked mutations in PLCγ1 and PLCγ2 are implicated in weakening of the autoinhibitory interface, resulting in unmasking of membrane-interacting surfaces and in higher PLC activity [10,23]. Consistent with this, the effect of such mutations is more readily observed in the assay where substrate is incorporated in lipid vesicles [23]. We here compared the WT and lipase-dead proteins with the PLCγ1^S345F^ variant (mutation at the TIM barrel/sPH interface) and PLCγ1^D1165H^ and PLCγ2^M1141K^ variants (mutations at the C2/cSH2 interface) (Supplementary Figure S3B). Additionally, the PLCγ2^P522R^ variant, not implicated in direct effects on autoinhibition, was included (Supplementary Figure S3B). While an increase in PLC activity was observed for PLCγ1^S345F^, PLCγ1^D1165H^ and PLCγ2^M1141K^ variants in the assay using lipid vesicles, the activity of the PLCγ2^P522R^ variant was comparable with the PLCγ2^wt^ (Figure 4A and B). Uniquely, an increase in PLC activity for the PLCγ1^S345F^ variant was also observed in the assay where substrate is hydrolysed in solution (Figure 4A, left panel).

To further understand possible mechanistic differences resulting in activation of the PLCγ1^S345F^ variant, we obtained a crystal structure for PLCγ1^S345F^ and performed atomistic molecular dynamics (MD) simulations for membrane lipid interactions (Figure 4C, Supplementary Figure S4 and Supplementary Table S1). Overall, although the PLCγ1^S345F^ structure in this crystal form (PDB ID: 9QB7) (Figure 4C, middle) is highly similar to the structure of the PLCγ1^wt^ (PDB ID: 7Z3J), there are notable differences. The major difference appears on the accessible area near the mutation which is decreased for the PLCγ1^S345F^ variant, while in parallel the hydrophobicity of the surface is increased. The mutation is located at the rim of the active site opening of the TIM barrel, involved in interactions with the membrane lipids, including its substrate. This feature, also known as a hydrophobic ridge, is substantially enhanced by the S345F mutation (Figure 4C, left panels). It is also possible that an increase in hydrophobicity in this region facilitates interactions with the hydrophobic part of the substrate mimetic (aldol-518 myo-inositol-1-phosphate) used in solution, contributing to higher PLC activity observed in assay 2 (Figure 4A, left panel). Further assessments by MD simulations of PLCγ1^S345F^ interactions with membrane lipids show that F345 is near the lipid tail of the active site bound PtdIns(4,5)P_2_ and that it interacts extensively with the lipid tails and cholesterol in neighbouring lipids in the vicinity of the active site (Supplementary Figure S4A).

Like PLCγ1^D1165H^ and PLCγ2^M1141K^ variants affecting the C2/cSH2 interface, the PLCγ1^S345F^ variant also affects the sPH/TIM autoinhibitory interface (Figure 4C, right panels). In an inactive form of the PLCγ1^wt^ residue S345 plays a key role in autoinhibition. In PLCγ1^S345F^, the interaction between S345 and Y509 is lost. However, the aromatic ring of F345 interacts with the side chain of I494 in the sPH domain *via* a CH-π bond. Similarly, within the sPH/TIM interface of the PLCγ1^S345F^ variant, there are other changes that could result in a loss or in a formation of new interactions between the residues in the TIM barrel and the sPH domain (Supplementary Figure S4B). Based on the experimental data suggesting the higher exposure of membrane interacting surfaces in the PLCγ1^S345F^ variant [11], it seems that an overall effect is weakening of the sPH/TIM autoinhibitory interface.

Subsequently to the characterization related to activation (Figure 4A–C), we also tested different disease-linked variants for inhibition by the nucleotides and observed a trend towards weaker inhibition regardless of possible mechanistic differences (Figure 4D). The largest differences were found for the PLCγ1^S345F^ and PLCγ1^D1165H^ variants compared with the PLCγ1^wt^ when inhibited by ADP (about 5–7-fold higher IC_50_ values). In cell-based assays, all tested variants are characterised by higher PLC activity [10,23], further supporting findings from structural insights suggesting that the nucleotides preferentially bind to an inactive form of PLCγ where autoinhibitory interactions, overlapping with the nucleotide binding site, are fully engaged. Unlike the PLCγ1^S345F^ variant (Figure 2D), the effect of disease-linked mutations on inhibition by nucleotides seems to be indirect, secondary to structural perturbations.

### Factors affecting inhibition by ATP and inhibition of PLCγ isozymes by different nucleotide compounds

**Figure 5:**
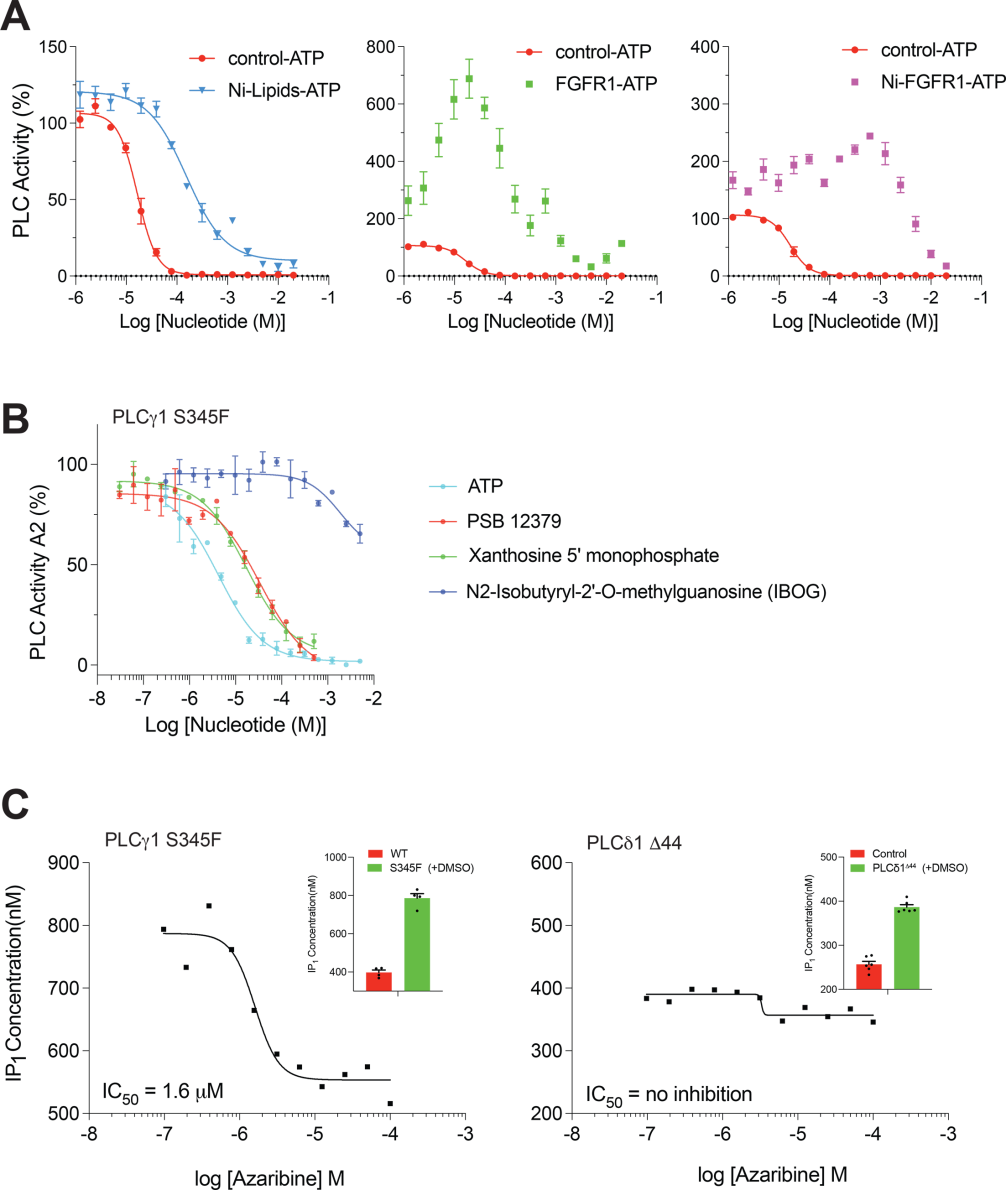
Several factors relevant to the cellular context affect ATP inhibition of PLCγ1 and synthetic nucleotide analogues and nucleotide pro-drugs can inhibit PLCγ1 *in vitro* and in cell-based assays. (**A**) Inhibition of PLCγ1^wt^ by ATP using the liposome embedded XY-69 (assay 1, **A1**) in the presence or absence of 10% Ni-lipids (left panel), the presence or absence of the FGFR1 kinase domain (middle panel) or the presence or absence of both 10% Ni-lipids and FGFR1-KD (right panel). Data are from three replicates and represents the mean and SD (**B**) The inhibition of PLCγ1^S345F^ mutant by ATP and various Nucleotide analogues from the MedChemExpress Nucleotide Compound Library. Data are shown as the means and SD of at least three experimental replicates and fit with an inhibitor vs. response (variable slope) equation in Graphpad Prism. (**C**) Dose–response curves showing the effect of Azaribine on the activity of indicated PLC isozymes expressed in stable cell lines. The inset shows the maximum IP_1_ concentration in the stable cell lines (green) compared with controls (red). Dose–response curves have been fitted with an inhibitor vs. response (variable slope) in Graphpad Prism and the IC_50_ shown on the graph.

One important aspect of PLCγ inhibition by nucleotide compounds in a cellular setting is their endogenous concentration, typically 1–10 mM for ATP [the average concentration about 4 mM [24]], that would be several fold in excess of a level sufficient to keep PLCγ in an inactive state. Corresponding concentrations of ADP are 10–100-fold lower, making ATP the more relevant ligand in this context. In cells, the activated PLCγ enzymes, either by physiological tyrosine phosphorylation or by mutations, interact with negatively charged membrane structures. These charged surfaces could repel negatively charged nucleotides and contribute, together with disruption of autoinhibition, to reduction in nucleotide/PLCγ interactions. To assess these possibilities, we first tested the effect of immobilisation of PLCγ1 to liposomes on the inhibition of PLC activity by ATP. It has been previously shown that immobilisation by His-tag/Ni-lipid interactions (achieved at 10% Ni lipids) enhanced PLC activity of PLCγ1 compared with a non-immobilised control [25], using similar conditions as in our experimental set up (Supplementary Figure S5A). Notably, this proximity of PLCγ1 to membrane structures prevented or, at higher concentrations, reduced inhibition by ATP (Figure 5A left panel; Supplementary Figure SB). We have also tested the PLCγ1 PLC activity in the presence of the intracellular portion of FGFR1 under conditions where the PLCγ1 directly binds to and is phosphorylated by the FGFR1-kinase [10]. With increasing ATP concentrations, contributing to phosphorylation/activation and to inhibition, an enhanced PLC activity compared with the control was observed (Figure 5A, middle panel). When combining the membrane proximity with the activated state of phospho-PLCγ1, representing conditions in stimulated cells, a clear reduction in nucleotide inhibition was observed over a range of ATP concentrations, including physiological levels (Figure 5A, right panel).

We further considered that other nucleotide compounds can also have an inhibitory effect *in vitro* and an ability to act at physiological concentrations of ATP in cell-based assays. Nucleotide compounds have been successfully developed into drugs (e.g. FDA-approved Valacyclovir) and many analogues are currently in clinical trials [26,27]. To test the possibility that in addition to ADP and ATP other nucleotide compounds can inhibit PLCγ enzymes, including their disease-linked variants, we tested a nucleotide library of about 500 compounds *in vitro* and subsequently selected a subset suitable for a cell-based assay (Figure 5B and C and Supplementary Figure S6 and S7). We identified compounds that inhibited PLCγ1^wt^ and, to a similar extent, or more potently, the disease-linked PLCγ1^S345F^ variant but had no effect on the PLC activity of PLCδ1^wt^ when tested *in vitro* (Supplementary Figure S6). As illustrated for several compounds (PSB 12379, Xanthosine 5’ monophosphate and IBOG), their efficacies for inhibition of PLCγ1^S345F^ vary (Figure 5B). The chemical structures of these nucleotides are presented in Supplementary Figure S7. Using a fluorescently labelled ATP analogue, we also demonstrated that the PSB 12379 compound competed with its binding (Supplementary Figure S7), suggesting binding to the common or overlapping site with the ADP/ATP ligands illustrated in Figure 2.

Using a cell-based assay that we previously established for studies of the PLCγ1^S345F^ variant [28], we also tested the efficacy of cell-permeable nucleotide prodrugs. As shown (Figure 5C left panel and Supplementary Figure S6C), one of the prodrugs, azaribine, potently inhibited PLC activity of PLCγ1^S345F^. This compound (the chemical structure shown in Supplementary Figure S7) had no effect on PLC activity of PLCδ1 in a stable cell line expressing this isoform from another PLC family (Figure 5C, right panel). These data suggest that nucleotide-based modalities can be developed to selectively inhibit PLCγ enzymes and their disease-linked variant not only *in vitro* but also in cells. Development and optimisation of such selective inhibitors and their detailed characterisation, including precise mapping of their binding sites, requires further extensive efforts.

## Discussion

Despite the recognition of PLCs as important components in the regulation of diverse biological functions and dysregulation in disease, the development of validated pharmacological tools and drug discovery efforts targeting PLC isozymes from different families has proved challenging. Focusing on two isozymes comprising the PLCγ family, PLCγ1 and PLCγ2, we here established the key characteristics of inhibition and binding of nucleotides, namely, ADP and ATP. Our studies support the selective inhibition of the PLCγ family and highlight an allosteric binding site with potential implications for physiological regulation of these enzymes and, importantly, for their targeting by synthetic nucleotide analogues.

Several lines of evidence presented here support binding of inhibitory nucleotide ligands to an allosteric site in PLCγ. Using HDX-MS, molecular docking and site-directed mutagenesis for the PLCγ1/ADP binding, this site is mapped to the interface between the TIM barrel from the PLC-core, common to all PLC isozymes, and the sPH domain from the PLCγ specific array (γSA) (Figure 2). Despite its proximity to this site, it is clearly distinct from the PLC active site in the central part of the TIM barrel. In addition, we have shown the requirement for both the PLC-core and γSA for the full nucleotide inhibition and selectivity for PLCγ isozymes (Figure 3). Direct kinetic measurements also support allosteric, non-competitive inhibition (Supplementary Figure S2).

**Figure 6:**
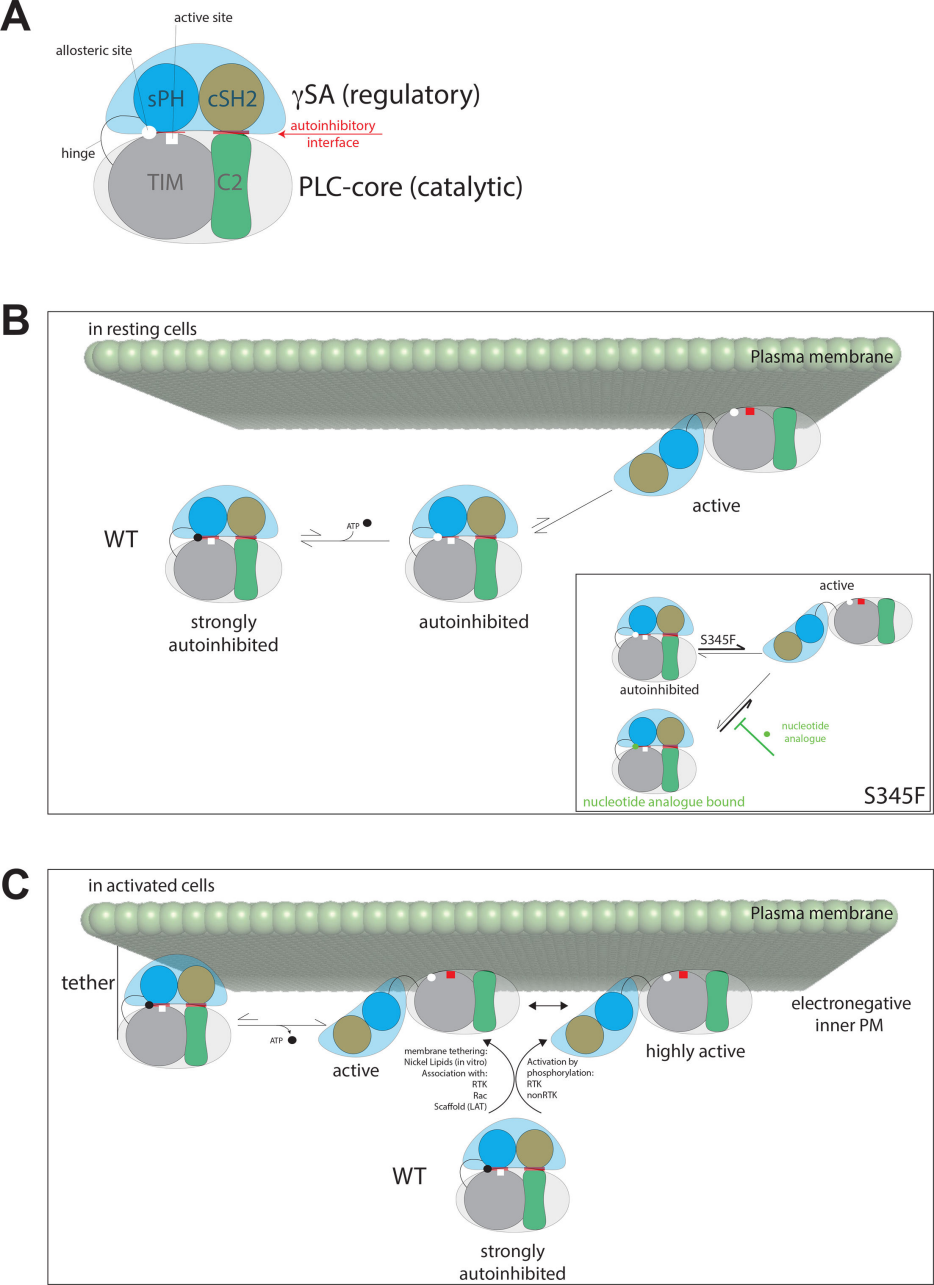
Implications of nucleotide inhibition on mechanistic models for autoinhibition, allosteric regulation and activation of PLCγ. (**A**) A cartoon representation of PLCγ in its autoinhibited form that summarises the findings of this work and previous reports. Two key parts are shown, a PLC core, consisting of nPH, EF hands, TIM barrel and C2 domain (only TIM and C2 are outlined) and the specific array (γSA) including regulatory domains sPH, nSH2, cSH2 and SH3 (only sPH and cSH2 are outlined). Interactions between these domains lead to an autoinhibitory interface that is higher affinity between the C2/cSH2 than the sPH/TIM. The active site and the location of the allosteric site are highlighted. (**B**) An equilibrium exists between autoinhibited and active PLCγ that strongly favours the autoinhibited form in non-stimulated cells. Binding of nucleotide at the allosteric site could act like ‘molecular glue’ and strengthens the autoinhibitory interaction between TIM and sPH. The interactions in the autoinhibitory interfaces and nucleotide together lead to a strongly autoinhibited enzyme. Inset: Certain mutations, such as S345F, cause a shift in the activity equilibrium (thick arrow) through their effect on the autoinhibitory interface and/or substrate interaction. The effect of these pathogenic mutations may be reversed through the application of novel nucleotide analogues (thick green blunt arrow). (**C**) With an IC_50_ for nucleotide in the low micromolar range and the cellular nucleotide concentration in the low millimolar range, how can PLCγ ever be active in cells? Factors such as proximity of negatively charged membrane and activation by phosphorylation that affects the binding site, reduce the nucleotide inhibition. Therefore, under such conditions (in stimulated cells) where the PLCγ is translocated to the PM through interaction with RTK, Rac, LAT scaffolds or other factors and/or becomes phosphorylated, the inhibitory effect of ATP and the autoinhibitory interaction of the γSA are overcome. In these instances, the equilibrium between autoinhibited and active PLCγ is shifted strongly to the active form.

The location of the nucleotide binding site, together with the analyses of inhibition in different PLC assays *in vitro*, further suggests possible molecular mechanisms for this allosteric inhibition. The current model for activation of PLCγ isozymes, following cell stimulation, outlines a shift of equilibria from an autoinhibited, inactive enzyme towards an active form, resulting from a large-scale conformational change and characterised by a release of autoinhibition and exposure of the membrane interaction surfaces [1,3] (Figure 6). Considering that the nucleotide binding site overlaps with one of the main autoinhibitory interfaces [10,11,23], stabilisation of autoinhibition by nucleotide binding would result in inhibition of PLC activity. Indeed, there is a notable inhibition of PLCγ activity in the PLC assay using substrate incorporated into lipid structures (assay 1) (Figure 1) where the activity is largely determined by the extent of the release of autoinhibition and availability of the membrane-interaction surface [13,23] (Supplementary Figure S1). However, substantial inhibition is also observed in a PLC assay using a small substrate mimetic that is hydrolysed in solution (assay 2) (Figure 1). This type of PLC assay can readily identify competitive (orthosteric) and canonical allosteric inhibitors directly affecting the active site. It is therefore possible that in addition to stabilising autoinhibition, nucleotide binding could allosterically impact on the adjacent active site in the TIM barrel.

Allosteric regulation is pervasive in nature and although allosteric modulators for therapeutic interventions are rare, the advantages of targeting such sites are widely recognised [29–31]. Accordingly, identification of an allosteric site in PLCγ has implications for understanding its physiological regulation as well as for new directions in drug discovery. Within a cellular context, the high concentrations of nucleotides such as ATP (1–10 mM) suggest that activation of PLCγ isozymes would be difficult to achieve owing to their inhibitory effect in the low micromolar range (Figure 1). However, as supported by data shown in Figure 5A, there are two possible mechanisms for overcoming nucleotide inhibition. Firstly, PLCγ tyrosine phosphorylation impacts on the nucleotide binding site, greatly reducing the inhibition. Secondly, PLCγ membrane recruitment and proximity of the enzyme to negatively charged membrane surfaces could facilitate the dissociation of bound electronegative ATP from the PLCγ protein. A model depicting the contribution of ATP binding to stabilisation of PLCγ autoinhibition and its reversal following cell stimulation is shown in Figure 6. Although further understanding of PLCγ regulation by ATP or other endogenous factors in cells requires more extensive studies, our findings expand upon the complexity of multiple interactions, including kinases, adapter proteins, small GTP-ases and nucleotides, that control the activity status of these enzymes.

Efforts to exploit allosteric sites in drug discovery have been stimulated by several potential advantages compared with orthosteric inhibition of the active site [31,32]. As is the case with families of PLC isozymes, the active sites are often highly conserved across the families. Allosteric sites are far less conserved, suggesting that achieving inhibitor selectivity is more likely. Our data showing selectivity of allosteric inhibition by nucleotides for PLCγ, compared with representative isozymes from two other important PLC families (Figure 3), illustrates this general concept. The other advantage of targeting allosteric sites, compared with active sites, is related to dynamic changes in proteins and higher flexibility that characterises allosteric sites, allowing for fine-tuning [29,31]. To achieve selective inhibition that would be applicable to a disease context, PLCγ inhibitors, exploiting the allosteric nucleotide binding site and its likely flexibility, would need to be effective in cells despite high concentrations of endogenous nucleotides and capable of targeting gain-of-function variants at least with similar potency as the WT proteins. Many gain-of-function variants of PLCγ1 and PLCγ2 discovered in diverse pathologies increase PLC activity by compromising autoinhibition to various degrees [10,23]. Additionally, other factors can contribute to higher PLC activity, as revealed by the crystal structure of PLCγ1^S345F^, an important, very frequent variant discovered in T-cell lymphomas (Figure 4C). This finding also suggests that despite a general trend to decrease sensitivity to nucleotides (Figure 4D), the effect of individual mutations on the allosteric site could differ in terms of magnitude and mechanism.

Focusing on the PLCγ1^S345F^ variant and the criteria outlined above, we further tested a library of synthetic nucleotide analogues for their suitability related to a disease relevant context (Figure 5B and C; Supplementary Figure S6). This resulted in the identification of compounds that inhibited the PLCγ1^S345F^ variant with similar or even higher potency than the PLCγ1^WT^ *in vitro,* while retaining selectivity compared with a member of another PLC family (Figure 5B; Supplementary Figure S6). Furthermore, a related pro-drug compound showed strong inhibition of the PLCγ1^S345F^ variant in a cell-based PLC assay, again retaining PLCγ family selectivity (Figure 5C). These findings suggest that further analyses and development of various nucleotide analogues, or other compounds targeting this allosteric site, could generate selective inhibitors of PLCγ with a potential to facilitate studies not only of their physiological functions and disease mechanisms but also to inform new efforts in drug discovery for these clinically important targets.

## Materials and methods

### Constructs and cloning

All protein expression work (except crystallography) utilised human PLCγ1 or PLCγ2 cloned in the pTriEx4(Gateway) vector that has been described previously [10]. These clones express in bacteria and mammalian cells and contain an N-terminal His-tag and a C-terminal Myc-tag. All mutants (point mutations and deletions) were generated using polymerase chain reaction with the site-directed mutagenesis kit-QuikChange II (Agilent). The open reading frames of all constructs were fully sequence verified prior to use. For crystallography experiments, the rat PLCγ1 clone described in [11] was utilised after creating an S345F amino acid substitution. The kinase domain of fibroblast growth factor receptor 1 (FGFR1) was utilised as outlined in [33].

For generating HEK cell lines that stably express PLC variants, an in-house Gateway compatible, Lenti viral vector was generated using In-Fusion cloning (Takara Bio) following manufacturer’s protocols. The vector backbone consisted of pLenti PGK Puro DEST (Addgene 19068). A KOZAK sequence, Avi-tag and His-tag (KAH cassette) was inserted between the PGK promoter and the attR1 vector regions. An IRES2 EmGFP cassette was inserted between the attR2 and WPRE regions. Both cassettes were PCR amplified from vector pHR-CMV-TetO2_3C-Avi-His6_IRES-EmGFP (Addgene 113888). Finally, a BirA ORF was cloned in-frame upstream of the EmGFP, which would express as a BirA-EmGFP fusion protein. The resulting Gateway compatible destination vector was used in the LR reaction (Thermo Fisher) with pENTR207 clones containing the PLCγ1^S345F^ or an activated version of PLCδ1, where the amino acids 445–487 had been deleted (PLCδ1Δ^44^), to generate their respective expression clones ready for preparing stable cell lines in HEK.

### Protein expression

All proteins were expressed in the phage resistant *Escherichia coli* strain, T7 Express *lysY/I^q^* (New England Biolabs). Cells transformed with the relevant plasmid were grown in 2xYT media containing 50 mg/ml ampicillin at 37°C until they reached an optical density at 600 nm of 2.0. Cells were then cooled to 15°C for 1 hour before the addition of 100 µM isopropyl-b-D-thiogalactopyranoside and allowed to express overnight. Cells were harvested by centrifugation and stored at −20°C until use.

### Protein purification

Cells were resuspended in lysis buffer [25 mM tris.Cl, 250 mM NaCl, 40 mM imidazole, 10 mM benzamidine, 1 mM MgCl_2_, 0.1 mM CaCl_2_ (pH 8.0)] containing protease inhibitors (1 EDTA free Roche tablet per 50 ml buffer). Cells were incubated at 10**°**C with constant shaking until fully resuspended. Cells were then lysed by being passed twice through a cell disruptor (EmulsiFlex-C5 High pressure homogeniser). Cell lysate was then centrifuged for 1 hour at 18,000 rpm in a Beckman JA-25.50 rotor at 4°C.

Lysate was loaded onto a 5 ml HisTrap column on an Akta Explorer system (Cytiva) previously equilibrated in His buffer A [25 mM tris.Cl, 500 mM NaCl, 40 mM imidazole, 1 mM TCEP (pH 8.0)]. The column was washed for 20 column volumes in His buffer A. Bound protein was eluted through the addition of His buffer B [25 mM tris.Cl, 500 mM NaCl, 500 mM imidazole, 1 mM TCEP (pH 8.0)]. Eluted protein was then dialysed overnight in SnakeSkin® dialysis tubing (Thermo Scientific) in dialysis buffer [25 mM tris.Cl, 20 mM NaCl, 1 mM EDTA, 1 mM TCEP, 0.1 mM EGTA, 10% glycerol (pH 8.0)] with gentle stirring overnight at 4°C. For the rat crystallisable protein, TeV protease was added during dialysis to a final ratio of 1:50 TeV:PLCγ1.

Subsequently, the dialysed protein was applied to a 5 ml HiTrap Heparin HP column (Cytiva) equilibrated in Heparin buffer A [25 mM tris.Cl, 20 mM NaCl, 1 mM TCEP (pH 8.0)]. The column was washed with 4 column volumes of Heparin buffer A. Protein was eluted in 10 ml fractions by a NaCl gradient over 20 column volumes through the addition of Heparin buffer B [25 mM tris.Cl, 1 M NaCl, 1 mM TCEP (pH8.0)]. Finally, fractions containing PLCγ were injected onto a HiLoad 26/60 Superdex 200 (Cytiva) column equilibrated in gel filtration buffer [25 mM HEPES.NaOH, 150 mM NaCl, 2 mM TCEP, 5% glycerol (pH 7.5)] and eluted with an isocratic gradient. For proteins to be crystallised, the crystal gel filtration buffer consisted of [25 mM HEPES.NaOH, 150 mM NaCl and 2 mM dithiothreitol (pH7.5)].

Monomeric proteins were concentrated using Amicon Ultra-15 centrifugal filtration units (Merck), generally to 2–5 mg/ml or 25 mg/ml for the crystal construct. Following concentrating, proteins were aliquoted, snap-frozen in liquid nitrogen and stored at −80°C until use. Purification of the FGFR1 kinase domain is outlined in [33].

### Nuclear magnetic resonance

Protein was buffer exchanged into phosphate buffered saline (pH 7.5) prepared in deuterium oxide. A 10 mM stock of ultrapure ADP was purchased from Promega. NMR spectra were acquired at 293 K in a Bruker Avance Neo 600 MHz spectrometer equipped with QCI-F cryoprobe and operating Topspin 4.2. Saturation transfer difference NMR experiments (stddiffesgp.3) [20] were acquired with 2 s saturation at 0.75 and 50 ppm using 50 ms Gaussian pulses. For the determination of *Kd*, ADP was added to the protein at ligand excesses from 5- to 100-fold. The protein concentration was 10 µM. STD amplification factors, $AF=\frac{{\left[L\right]}_{0}}{{\left[P\right]}_{0}}\cdot \frac{I_{ref}-I_{sat}}{I_{ref}}$ , were measured and fitted to a hyperbolic dose-response model to determine the dissociation constant for the protein/ADP interaction.

### Microscale thermophoresis

Proteins were diluted to 200 nM in MST buffer [50 mM Hepes.KOH, 70 mM KCl, 5% (v/v) glycerol, 0.05% (v/v) Tween-20, 2 mM DTT (pH 7.5)] and subsequently labelled by adding an equal volume of 100 nM 2^nd^ generation RED-Tris-NTA labelling dye and incubating for 30 minutes. A 16-fold serial dilution of ATP or ADP was prepared in MST buffer with a maximum concentration of 25 mM. An equal volume of labelled protein was added to each ligand concentration and the mixture taken up into a standard MST capillary. Samples were measured in a Monolith NT.115 pico instrument (Nanotemper) using the Nano-RED channel. Excitation power was set to 5% and MST power set to high. All experiments were performed at 25°C. All data were analysed using the manufacturer’s MO.Control software and repeated at least three times with representative experiments shown.

### Fluorescence polarisation assay

Experiments were performed in black small volume 384-well plates (Greiner) on a Pherastar FS microplate reader (BMG Labtech) utilising an FP module with Excitation 485 nm and Emission 520 nm. Initial experiments determined a suitable concentration of the fluorescent ATP analogue, 2,4,6-trinitrophenol ATP (TNP-ATP, Jena Bioscience) that generated a large dynamic range in the FP setup. The optimal concentration was determined to be 1 mM with a gain of 500; the optimisation was necessary because different ATP analogues such as TNP-ATP have different binding affinities compared with ATP. PLCγ1^S345F^ protein was titrated against the fixed concentration of TNP-ATP and the FP data fit in GraphPad Prism using the One site Total binding model. Subsequently, the highest concentration of protein with TNT-ATP was titrated with increasing concentrations of a synthetic ATP analogue, PSB12379, that had been previously solubilised in DMSO. Equal amounts of DMSO only were added in a parallel FP experiment. Generated data was also fit in GraphPad Prism using the One site -Total binding model. Each experiment was performed in triplicate and the data presented as the mean and SD.

### Hydrogen deuterium exchange mass spectrometry

#### HDX-MS sample preparation

HDX reactions comparing apo hPLCγ1 to hPLCγ1 incubated with ADP were carried out in a 15 µl reaction volume containing 10 pmol of hPLCγ1 (0.67 µM) and 10 µM of ADP and 1.67% DMSO. The exchange reactions were initiated by the addition of 10.0 µl of D2O buffer (25 mM HEPES pH 7.5, 150 mM NaCl, 2 mM TCEP 92.25% D_2_O (V/V)) to 5.0 µl of protein (final D2O concentration of 61.5%). Reactions proceeded for 3 s at 0°C, 30 s, 300 s and 3000 s at 20°C before being quenched with ice cold acidic quench buffer, resulting in a final concentration of 0.6M guanidine HCl and 0.9% formic acid post quench. All conditions and time points were created and run in independent triplicate. Samples were flash frozen immediately after quenching and stored at -80°C until injected onto the ultra-performance liquid chromatography (UPLC) system for proteolytic cleavage, peptide separation and injection onto a QTOF for mass analysis, described below.

#### Protein digestion and MS/MS data collection

Protein samples were rapidly thawed and injected onto an integrated fluidics system containing a HDx-3 PAL liquid handling robot and climate-controlled (2°C) chromatography system (Trajan), a Waters Acquity UPLC I-Class Series System, as well as an Impact HD QTOF Mass spectrometer (Bruker). The full details of the automated LC system were previously described [34]. The samples were run over an immobilised pepsin column (Affipro; Enzymate Protein Pepsin Column, 2.1 mm X 20 mm) at 200 µl/min for 3 minutes at 2°C. The resulting peptides were collected and desalted on a C18 trap column (Acquity UPLC BEH C18 1.7  µm column (2.1  ×  5  mm); Waters 186004629). The trap was subsequently eluted in line with an ACQUITY 300 Å, 1.7 μm particle, 100 × 2.1 mm BEH C18 UPLC column (Waters), using a gradient of 3-10% B (Buffer A 0.1% formic acid; Buffer B 100% acetonitrile) over 1.5 minutes, followed by a gradient of 10–25% B over 4.5 minutes, followed by a gradient of 25–35% B over 5 minutes, finally after 1 minute at 35% B a gradient of 35–80% B over 1 minute was used. Mass spectrometry experiments were acquired over a mass range from 150 to 2200 m/z using an electrospray ionisation source operated at a temperature of 200°C and a spray voltage of 4.5 kV.

#### Peptide identification

Peptides were identified from the non-deuterated samples of p110α using data-dependent acquisition following tandem MS/MS experiments (0.5 s precursor scan from 150 to 2000 m/z; twelve 0.25 s fragment scans from 150 to 2000 m/z). MS/MS datasets were analysed using FragPipe v18.0 and peptide identification was carried out by using a false discovery-based approach using a database of purified proteins and known contaminants [35–37]. MSFragger was utilised, and the precursor mass tolerance error was set to −20 to 20ppm. The fragment mass tolerance was set at 20ppm. Protein digestion was set as nonspecific, searching between lengths of 4 and 50 aa, with a mass range of 400 to 5000 Da.

#### Mass analysis of peptide centroids and measurement of deuterium incorporation

HD-Examiner Software (Trajan) was used to automatically calculate the level of deuterium incorporation into each peptide. All peptides were manually inspected for correct charge state, correct retention time, appropriate selection of isotopic distribution, and so on. Deuteration levels were calculated using the centroid of the experimental isotope clusters. Results are presented as relative levels of deuterium incorporation and the only control for back exchange was the level of deuterium present in the buffer (61.5%). Differences in exchange in a peptide were considered significant if they met all three of the following criteria: ≥4.5% change in exchange, ≥0.45 Da difference in exchange, and a *P* value <0.01 using a two-tailed Student t-test. Samples were only compared within a single experiment and were never compared with experiments completed at a different time with a different final D_2_O level. The data analysis statistics for all HDX-MS experiments are in Supplemental source data according to published guidelines [22]. The mass spectrometry proteomics data have been deposited to the ProteomeXchange Consortium via the PRIDE partner repository [38] with the dataset identifier PXD051153.

### Preparation of liposomes incorporating the XY-69 compound

All lipids used in this work were supplied by Avanti Polar Lipids. Some batches of liposomes were prepared with porcine brain phosphatidylethanolamine (PE), and some were prepared with synthetic PE. All following % composition details are expressed as (w/v). A standard liposome mix was generated as well as a mix containing nickel lipids.

The standard liposome components were designed to mimic a typical plasma membrane composition [39]: 49% PE (brain or synthetic), 1% phosphatidylinositol 4,5-bisphosphate (PIP_2_), 20% brain phosphatidylserine (PS), 15% phosphatidylcholine (PC), 10% cholesterol and 5% sphingomyelin. The nickel liposome mix consisted of 39% PE (brain or synthetic), 1% PIP_2_, 20% brain PS, 15% PC, 10% cholesterol, 10% nickel lipid and 5% sphingomyelin.

The synthetic fluorogenic PIP_2_ analogue XY-69 was added to a borosilicate glass tube and the aqueous solvent evaporated using a gentle stream of nitrogen gas. The above lipids were added to the tube and the organic solvents were likewise evaporated using a gentle stream of nitrogen gas. The lipids were dried under vacuum for >1 hour at room temperature. Lipids were resuspended in 20 mM Hepes.KOH (pH 7.4) and subjected to a freeze-thaw cycle between acetone on dry ice and a 42°C water bath followed by vortexing and probe sonication. The liposomes were subjected to 11 further freeze-thaw cycles. Finally, liposomes were extruded through a 100 nm filter five times and mixed in an equal volume of 2X assay buffer (see below). The final concentration of lipid within the liposome suspension was 2 mg/ml. Liposomes were either used immediately after preparation or were stored at −80°C.

### Measurement of PLC activity *in vitro*

#### Standard nucleotide inhibition of PLC activity

The effect of nucleotide inhibition on PLC activity was measured using either a membrane-based assay with the fluorescent substrate (XY-69) incorporated into liposomes or using a small soluble substrate, Aldol 518 myo-inositol-1-phosphate (Aldol). Both methods utilised the same PLC assay buffer [20 mM Hepes-KOH, 70 mM KCl, 3 mM EGTA, 2.97 mM CaCl_2_, 2 mM TCEP and fatty acid-free bovine serum albumin (FAF-BSA; 50 μg/ml) (pH 7.5)]. Both assays were performed in low volume black 384-well plates (Greiner Bio-One), were composed of a final assay volume of 20 μl and were monitored in a Clariostar plate reader (BMG Labtech). The Aldol assay consisted of 4 μl of a serial dilution of ATP/ADP in assay buffer, 8 μl of PLC protein (concentration varied between PLC variants), and 8 μl of Aldol 518 myo-inositol-1-phosphate (final concentration in assay, 50 μM). The excitation and emission wavelengths were 588–15 and 642–20, respectively.

The XY-69 assay contained 4 μl of a serial dilution of ATP/ADP in assay buffer, 4 μl of PLC protein (concentration varied between PLC variants), and 12 μl of XY69 liposomes (final concentration in assay 1.2 mg/ml). A CLARIOstar plate reader (BMG Labtech) was used to measure the production of the fluorescent product. The excitation and emission wavelengths were 485–15 and 520–20, respectively. Each experiment was performed in at least triplicate; in many cases, nine-fold replicates were generated. The enzyme progress curves were plotted for each concentration of nucleotide using Graphpad Prism software. The initial linear rate of product generation was calculated and converted to a % inhibition when compared with the rate of PLC activity in the absence of nucleotide. These % inhibition values were plotted against the logarithm of the nucleotide concentration in Graphpad Prism and the data fitted with the function (Inhibitor) vs. response–variable slope (four parameters). The resultant fit yielded the IC50 values for the nucleotide inhibition.

#### Elucidation of competitive or non-competitive inhibition of PLC by nucleotides

The standard XY-69 assay outlined above was used to determine the type of inhibition of PLCγ1^S345F^ by ATP. PLCγ1, with the final concentration of 10 nM, was used to test inhibition with three concentrations of ATP: 0 μM, 5 μM and 50 μM. For each ATP concentration, a ten-fold dilution of the XY69 substrate was used, with the highest concentration being 500 μM. The slopes of each curve for each ATP concentration were calculated using a non-linear fit in GraphPad Prism. The slopes were then plotted against the logarithm of XY-69 concentration to obtain the Lineweaver-Burk plot and therefore the *Km* and *Vmax* values. The change in the obtained *Km* and *Vmax* values across different concentrations of ATP allowed determination of the type of inhibition caused by ATP. Similarly, a known competitive inhibitor AlCl_3_ was used as a control in three different concentrations: 1 μM, 25 μM and 200 μM.

#### Elucidation of the effect of nickel lipids on PLC inhibition by nucleotides

The standard XY-69 assay format outlined above was repeated with the addition of 10% nickel lipids in the liposome mixture. The assay used PLCγ1^WT^ protein (final concentration 12.5 nM) that has an N-terminal His-tag that can interact and bind to the liposomes containing nickel lipids.

#### Elucidation of the effect of phosphorylation on PLC inhibition by nucleotides

The standard XY-69 assay outlined above was followed with the following changes. The PLC activity buffer also contained 10 mM MgCl_2_. The final concentration of PLCγ1^WT^ in the assay was 12.5 nM and also contained a 10-fold excess of the FGFR1 kinase domain. The ATP dilution series was first added to the assay plate, followed by the enzyme mix. The plate was incubated for 1 hour at room temperature to allow the FGFR to phosphorylate the PLCγ1. Subsequently, the standard XY-69 liposomes (with or without nickel lipids) were added, and the experiment monitored in the Clariostar as outlined above.

### Measurement of PLC activity in cells

#### Generation of HEK cell lines stably expressing PLC variants

Stable cell lines overexpressing PLCγ1^S345F^ or PLCδ1^Δ44^ were prepared as described in [28]. Briefly, lentiviral vectors were polyethyleneimine (PEI) transfected into HEK293 cells and selected with 2 mg/ml puromycin. Fluorescence-activated cell sorting (FACS) was used to select the top 10% of GFP-expressing cells. Stable cell lines were expanded and either used immediately in experiments or cryopreserved in liquid nitrogen.

#### Measurement of PLC variant activity in HEK stable cell lines

PLC activity in cell lines was measured using the IP-ONE assay according to the manufacturer’s instructions as described in [28]. Briefly, stable cell lines were plated in 96-well plates with 30,000 cells in 50 μl media. Nucleotide inhibitors were added in a further 50 μl media at final concentrations as stipulated in the figures. The following day, media were removed and replaced by media containing 50 mM LiCl. The cells were incubated for 1 to 2 hours before lysing and measuring the IP1 generated. GraphPad Prism was used to plot data and fit with the function (Inhibitor) vs. response–variable slope (four parameters). The resultant fit yielded the IC_50_ values for the nucleotide inhibition. Experiments were repeated at least three times and representative data are shown.

### Nucleotide library screening

A compound library containing 497 nucleotides, nucleosides and structural analogues was obtained from MedChemExpress (Cat. No.: HY-L044). Compounds were supplied in 384-well microplates as pre-dissolved solutions in DMSO or H_2_O (453 compounds at 10 mM in DMSO, 2 compounds at 2 mM in DMSO, 40 compounds at 10 mM in H_2_O, 1 compound at 2 mM in H_2_O, 1 compound at 3 mg/ml in H_2_O). A 30 μl of each solution was provided. For measuring the effect of each compound on PLC activity, the assay 2 (A2) was performed, as described in the main section of materials and methods, with 1 μl of each compound, 9.5 μl of PLC protein (concentration varied between PLC variants), and 9.5 μl of aldol 518 myo-inositol-1-phosphate (final concentration in assay, 50 μM) added to wells of low volume black 384-well plates (Greiner Bio-One). The assay 2 was performed for each compound with PLCγ1^WT^, PLCγ1^S345F^, PLCγ2^WT^ and PLCδ1^WT^. PLC protein + aldol substrate only, and PLC protein + 10 mM ADP (final concentration 0.5 mM) + aldol substrate were included in each plate as negative and positive controls respectively. After obtaining the activity curves, their slopes were calculated using the linregress function of the SciPy Python module. Using the obtained slopes, heatmaps were generated with the Seaborn Python module, where the minimum value was taken to be the average of the positive control slopes, while the maximum value was taken to be three times the average of the negative control slopes. Therefore, this allowed coverage of 0 to 300% PLC activity. From the generated heatmaps, compounds displaying PLCγ1^S345F^-selective inhibition were identified. Serial dilutions of selected compounds were created, and the assay 2 was repeated as above in triplicate with PLCγ1^WT^, PLCγ1^S345F^ and PLCδ1^WT^. IC_50_ values were generated from the resultant data as described in the main section of materials and methods.

### Crystallisation and crystallography data processing

#### Protein crystallisation

The purified rat PLCγ1^S345F^ was concentrated to 14 mg·ml^−1^. The protein was crystallised using the hanging drop vapour diffusion method on a 24-well VDX crystallisation plate (Hampton Research) screening conditions around the previously crystallised complex rat PLCγ1/IP_3_ (PDB ID 7Z3J). A crystal grown in 16% PEG 3,350 and 0.1 M Citric acid BIS-TRIS propane (CBTP) pH 7.0 was harvested and immersed for 10 s in a solution containing the precipitant mixture and 12% (v/v) 2-Methyl-2,4-pentanediol (MPD) and cryo-cooled in liquid nitrogen.

#### Data collection and refinement

A crystal of the rat PLCγ1^S345F^ was measured at the I04 beam-line (Diamond Light Source, UK) at 100 K and processed using the autoPROC package (Global Phasing Limited) using programs from XDS suite and CCP4 [40,41]. The crystal belonged to the *P 2_1_ 2_1_ 2_1_* space group with a solvent content of 51.8% corresponding to one protein molecule in the asymmetric unit. The structure was determined by simple rigid body refinement using the PHENIX suite [42] and the previously determined rat PLCγ1/IP_3_ (PDB ID 7Z3J) where waters and ligands were removed. Extra density at the residue 345 confirmed the successful substitution of serine to phenylalanine. The rigid-body refined model was further processed with consecutive iterations of TLS/maximum-likelihood refinement using phenix.refine and manual model inspections using COOT [43]. Solvent and ligand molecules were added during this process and the model converged to a final *R*_work_/*R*_free_ of 18.81/23.33% at a maximum resolution of 2.56 Å. Data collection and refinement statistics are summarised in Supplementary Table S1).

### Computational modelling

Computational simulations were carried out using the Maestro portal (Shrödinger, Inc.). The protein (rat PLCγ1) was prepared from the PDB ID 7Z3J using the ‘protein preparation’ wizard of Maestro. The software added hydrogens, adjusted the side chains using a pH of 7.4 and optimised side chains and charges using the OPLS4 energy minimisation protocol. The ADP molecule was prepared by LigPrep. Glide was used first to generate the receptor grids near the positions that were detected as binding sites from the HDX-MS analysis. For docking, the settings included extra precision (XP), without any constraints. Binding was evaluated based on the Glide G score. Parameters for the selected docking outcome: PLC residues = 367–386; number of ADP conformations = 32 (21); docking score = −7.99; Glide G score = −9.09; Glide Emodel = −62.42. The same methodology was applied when using ATP as a ligand.

### Atomistic MD simulations

Overall, the MD simulations for the PLCγ1^S345F^/membrane lipid interactions were performed as previously described [11], based on specific steps [44–49]. To model how the S345F mutant interacts with the membrane, all-atom molecular dynamics simulations were conducted using the CHARMM36 force field in GROMACS (version 2024.4) [44,45]. To generate the mutant *in silico,* we used the structure and atomic co-ordinates from the end point of previously published all-atom simulations of the PLCγ1 core domains bound to the membrane, with PI(4,5)P_2_ present in the active site [11]. The rotamers tool of ChimeraX (version 1.9) was used to mutate serine 345 from the previous simulation model to phenylalanine, selecting the rotamer predicted to be the most stable from the rotamer library [46]. The mutated system was relaxed by performing energy minimisation using the steepest descent method and then subjected to equilibration in the NPT ensemble with the protein backbone co-ordinates restrained, for 1 ns with a 1 fs timestep, using the Berendsen thermostat at 323 K, and the semi-isotropic Berendsen barostat at 1 bar [47]. 3 production simulation replicates were conducted each initialized from the equilibrated system, with velocities sampled from a Boltzmann distribution. Production simulations were run for 50 μs with 20 fs timestep, using the velocity-rescaling thermostat (323 K) and semi-isotropic Parrinello–Rahman barostat (1 bar) [48,49].

#### Datasets

The data for crystal structure [50], the HDX-MS mass spec data [51] and the MD simulation data [52] have been deposited and are available to access.

## Supplementary material

Online supplementary material 1

Online supplementary material 2

## Data Availability

All data required to evaluate the conclusions of this paper are present in the paper and/or the Supplementary Materials. The information for crystal structure is deposited with the Protein Data Bank (PDB) under accession number 9QB7. The HDX-MS mass spec data are available at the PRIDE depository with accession number PXD051153. MD simulation data are available in: https://doi.org/10.5518/1721. Additional data related to this paper may be requested from the authors.

## Abbreviations

DAG: diacylglycerol
HDX-MS: hydrogen deuterium exchange mass spectrometry
HTRF: homogeneous time-resolved fluorescence
HTS: high-throughput screening
IP_3_: inositol(1,4,5)trisphosphate
MST: microscale thermophoresis
PIP_2_/PI(4,5)P_2_: phospatidylinositol 4,5-bisphosphate
PLCγ: phospholipase C gamma
RTKs: receptors tyrosine kinases
