# Supplementary material for: Characterisation of an allosteric site in PLCγ enzymes and implications for development of their specific inhibitors
Source: Biochem J. 2025 Oct 16;482(20):1545–64. doi: 10.1042/BCJ20253358 (PMC12687421; doi:10.1042/BCJ20253358)
Supplement: Online supplementary material 1 [file bcj-482-20-BCJ20253358-s001.pdf]

Supplementary Materials for

Characterisation of an allosteric site in PLCγ enzymes and implications for development of their specific inhibitors

Tom D. Bunney *et al.*

Corresponding authors: t.bunney@ucl.ac.uk and m.katan@ucl.ac.uk

This PDF file includes:

Figs. S1 to S8
Table S1

Other Supplementary Materials for this manuscript include the following:

Data S1 (HDX-MS Source Data)

Fig. S1.

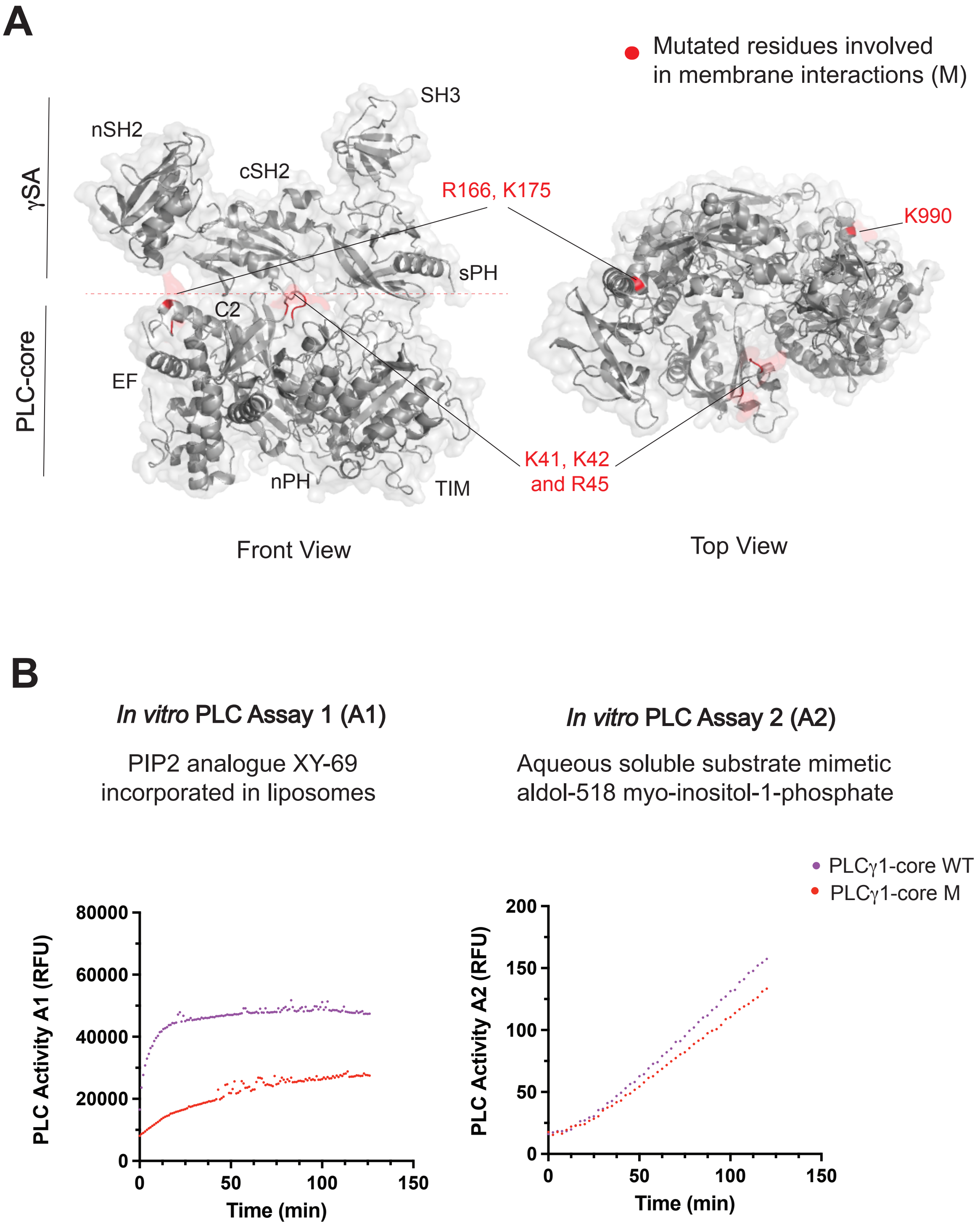

Fig. S1. **Characterisation of assays for measurements of PLC activity *in vitro*.** (**A**) Structure of an autoinhibited PLCγ1 (PDB 7Z3J), front view, with the individual dominants in the PLC-core and regulatory γSA labelled in black; red dotted line indicates interfaces between the PLC-core and γSA (left panel). Based on the model of PLCγ1 activation, the PLC-core/γSA interactions are compromised following stimulation, exposing surfaces on the PLC-core (shown as a top view) for membrane association (right panel). Residues highlighted in red have been implicated in membrane interactions and in the variant designated as PLCγ1-core M, mutated to alanine. (**B**) Comparison of the PLCγ1-core WT and PLCγ1-core M variants in two *in vitro* assays for measurements of PLC activity. Assay 1 (A1) monitors hydrolysis of a fluorogenic compound XY-69, an analogue of PI(4,5)P_2 (PIP2) that is incorporated in liposomes (left). Assay 2 (A2) uses aqueous soluble substrate mimetic, aldol-518 myo-inositol-1-phosphate, as a substrate hydrolysed in solution (right panel).

Fig. S2.

A

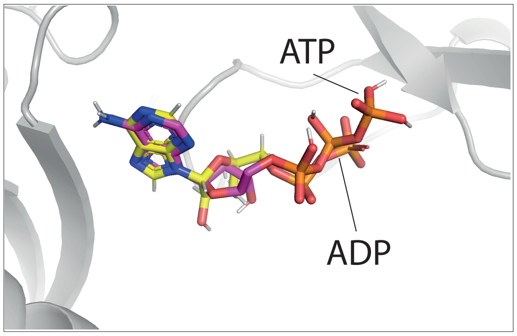

B

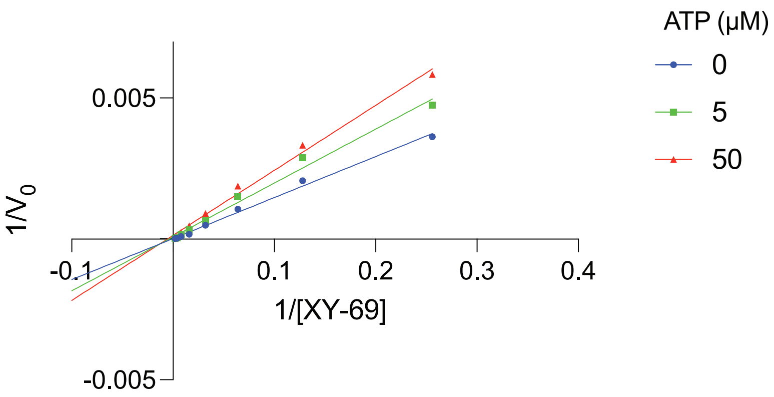

**Best-fit values / ATP concentrations (µM)**	0	5	50
slope	0.007241	0.008530	0.01019
1/slope	381.1	117.2	98.13
Y intercept: $1/V_{max}$	0.0006757	0.001075	0.001318
X-intercept: $-1/K_m$	- 0.09331	- 0.1260	- 0.1293

Fig. S2. Docking of ATP and kinetic analysis of inhibition by ATP. (**A**) Docking of ATP onto the structure of PLCγ1^{wt}. A docking outcome that locates the nucleotide at the TIM-sPH interface is shown and overlayed with the docked ADP presented in Figure 2C. (**B**) Linear representation (top) and best-fit values (bottom) for measurements obtained in the range (3.9 - 500 µM) of XY-69 concentrations, in the absence and presence of indicated concentrations of ATP.

Fig. S3.

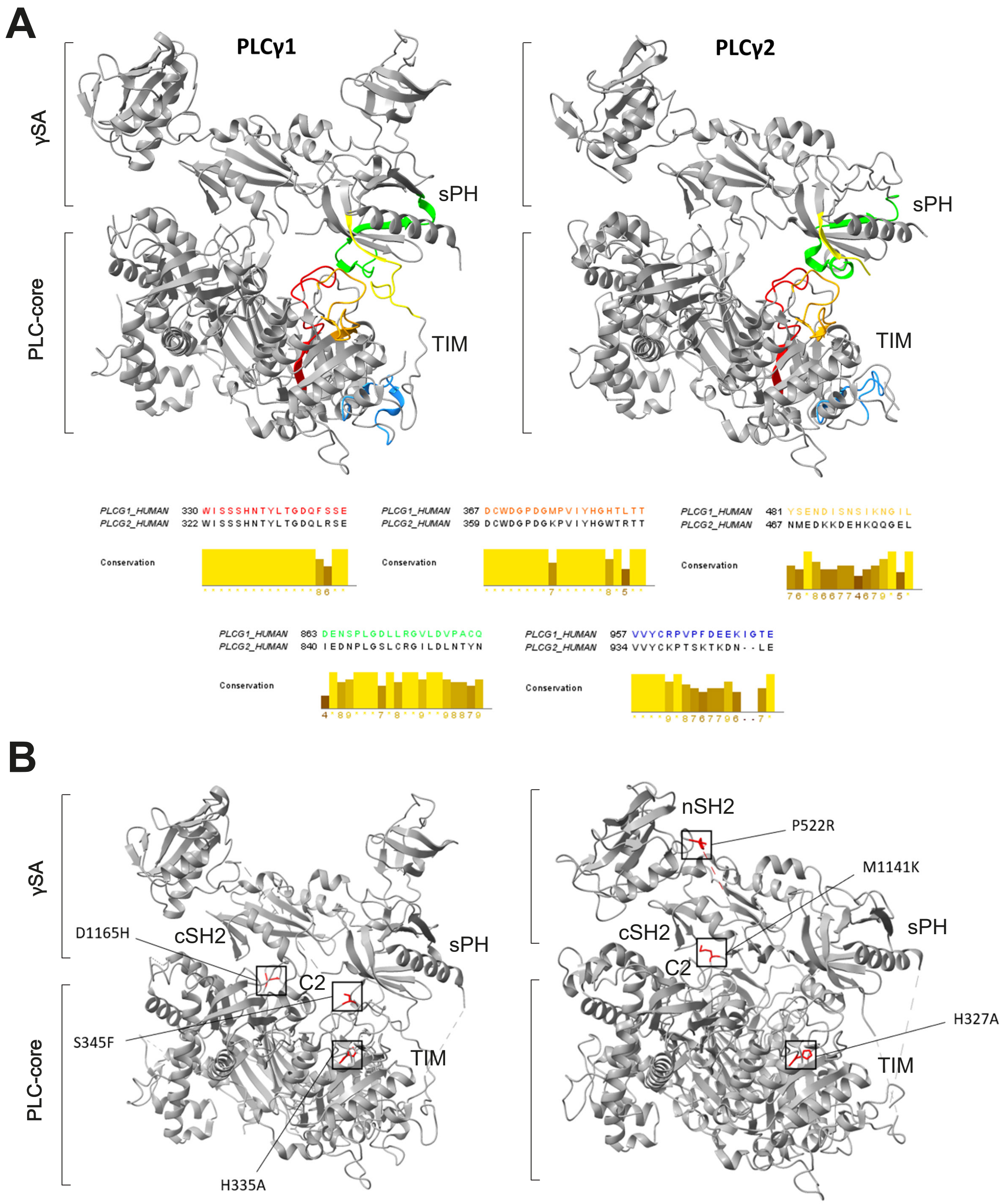

Fig. S3. **Comparison of PLCγ1 and PLCγ2 3D-structures**. (**A**) Similar structural organisation of PLCγ1 (7Z3J) and PLCγ2 (8JQG). Peptides in PLCγ1 with reduced HD-exchange following nucleotide binding at the sPH/TIM barrel interface (top left) and corresponding peptides in PLCγ2 (top right) are coloured in red, orange, yellow, green, or blue as indicated for each peptide. Sequence alignment and conservation of residues in these peptides between human PLCγ1 (P19174) and human PLCγ2 (P16885) is calculated and presented below. (**B**) Positions of residues mutated in disease together with the active site histidine, analysed in this study, are shown in red. Mutation PLCγ1 S345F is at the sPH/TIM autoinhibitory interface. Mutations PLCγ1 D1165H and PLCγ2 M1141K are at the cSH2/C2 autoinhibitory interface. Mutation PLCγ2 P522R is in the nSH2/sPH linker. Mutations PLCγ1 H335A and PLCγ2 H327A remove the catalytic histidine in the active site.

Fig. S4.

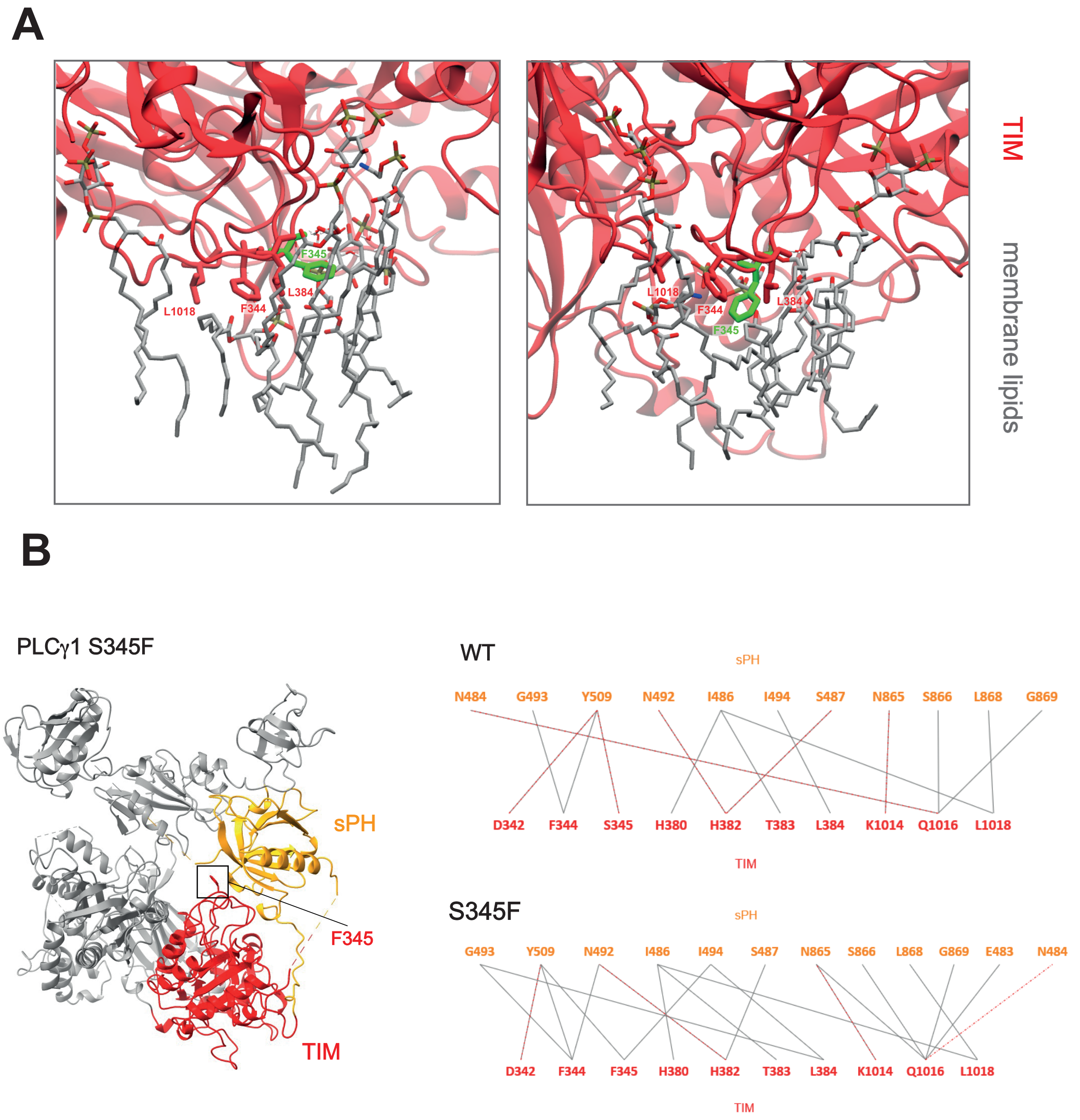

Fig. S4. **Structure of the PLCγ1 S345F variant**(**A**) Interactions of the hydrophobic ridge of the PLCγ1 S345F variant with the membrane. Two representative rotameric states of F345 observed during all-atom MD simulations are shown (left and right). F345 (green sticks) inserts into the membrane, and alongside other hydrophobic ridge residues (F344, L384 and L1018; red sticks) of the TIM-barrel domain (red), interacts with cholesterol and lipid tails (grey sticks, with O atoms shown in red and P atoms shown in brown) in the vicinity of the active site. (**B**) Effect of S345F mutation on autoinhibitory interface. Crystal structure of the PLCγ1 S345F variant (PDB ID: 9QB7) with the TIM-barrel shown in red and the sPH domain in orange (left). Schematic representation of interactions at the sPH/TIM autoinhibitory interface for the PLCγ1 WT and PLCγ1 S345F variant (right).

Fig. S5.

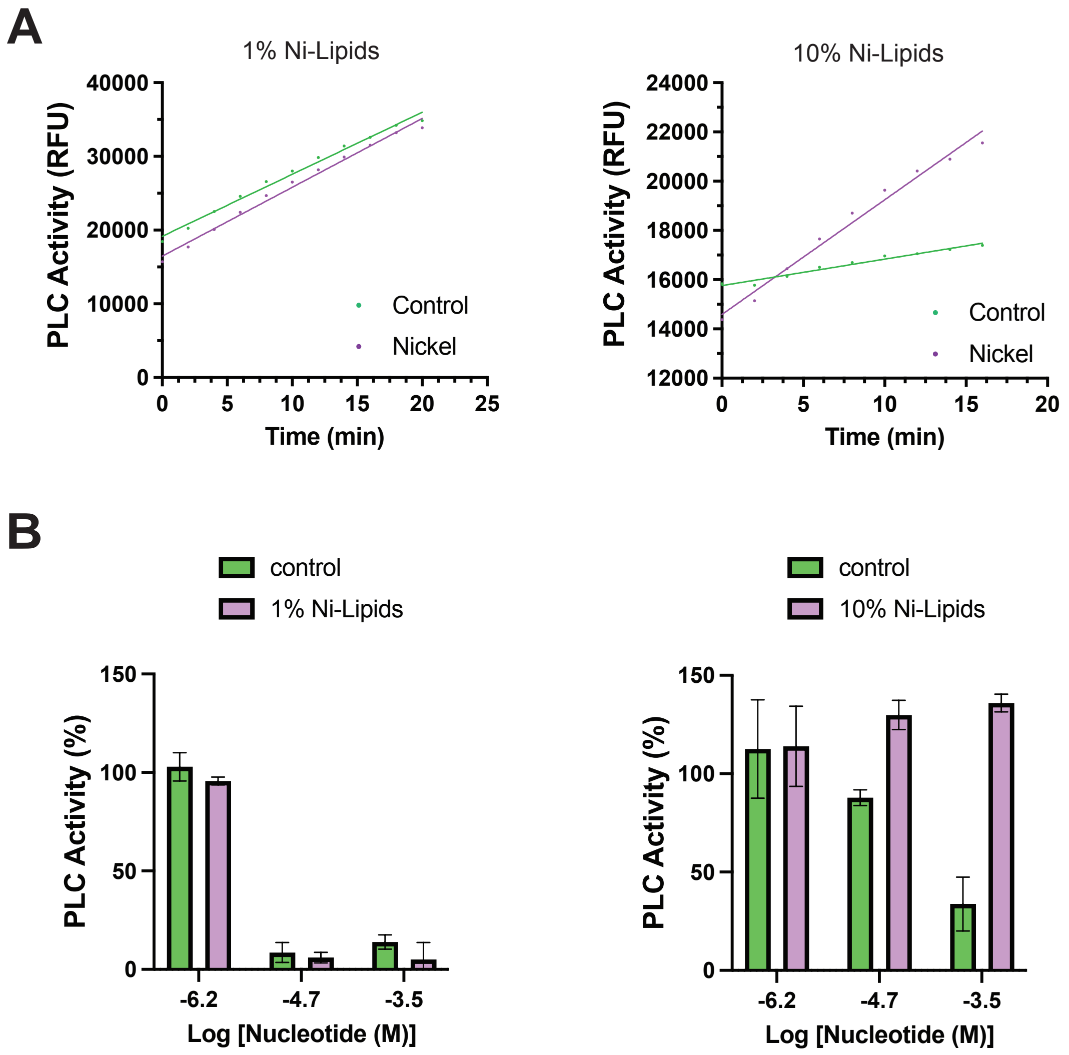

Fig. S5. **PLC activity and ATP inhibition of PLCγ1 immobilized on liposomes.** (**A**) PLC activity of the His-tagged PLCγ1 was measured in a modified assay 1 (A1) with inclusion of 1% (left) or 10% (right) of nickel lipids (Ni-lipids). (**B**) Inhibition of PLC activity by indicated concentrations of ATP in the same assay including 1% (left) or 10% (right) Ni-lipids.

Fig. S6.

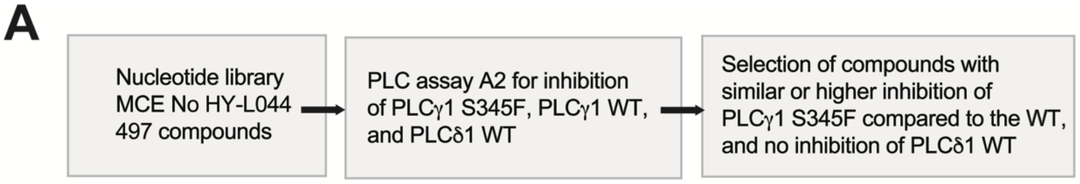

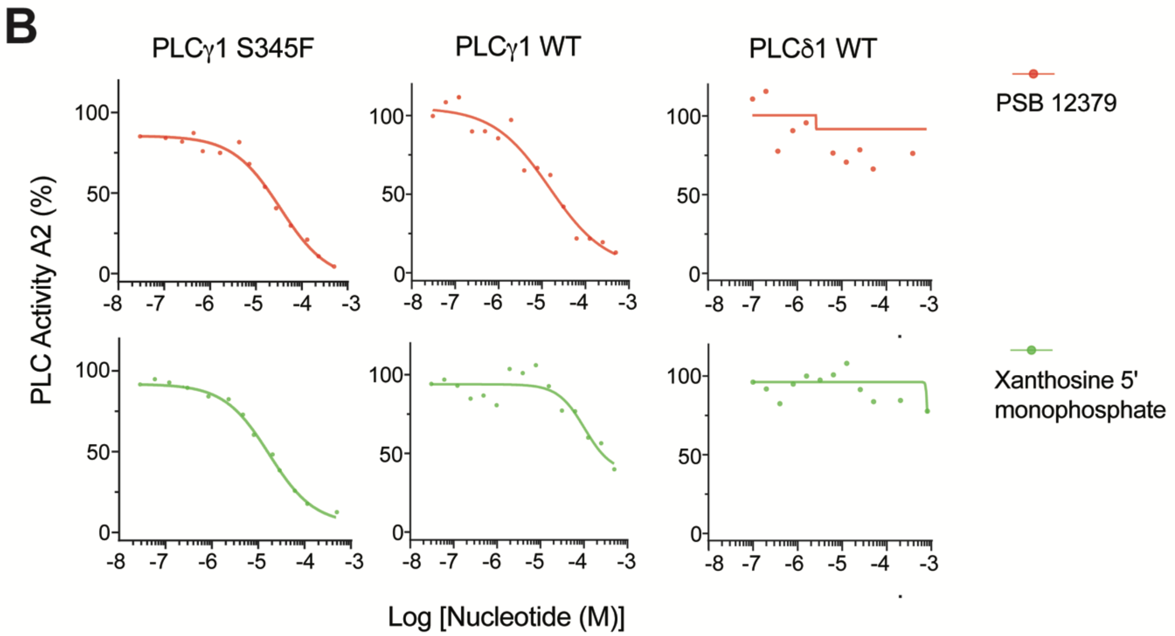

A2 IC_{50} (μM)		
PLC variant \ Compound	PSB 12379	Xanthosine 5' monophosphate
PLCγ1 S345F	34.0 ± 1.9	17.2 ± 1.4
PLCγ1 WT	14.9 ± 5.5	103.0 ± 32.3
PLCδ1 WT	No inhibition	No inhibition

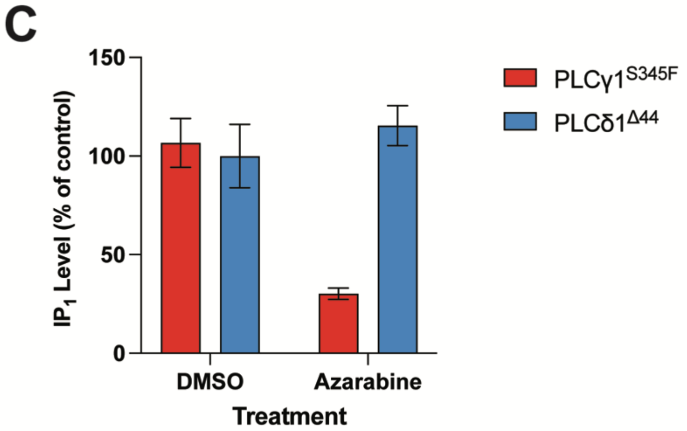

Fig. S6. **Inhibition of PLC variants by compounds from a nucleotide library.** (**A**) Outline of the screen. (**B**) Representative examples assessing inhibition of PLCγ1 S345F, PLCγ1 WT and PLCδ1 WT by two selected compounds: PSB 12379 (red) and Xanthosine 5' monophosphate (green) (top panels). A summary of IC_{50} values for the two compounds, obtained from 3 measurements, with the values presented as the mean and SD (bottom). (**C**)

Histogram showing the effect of 3 μM of the nucleotide pro-drug, Azarabine, on the turnover of IP_1 in stable cell lines expressing PLCγ1^{S345F} and PLC δ$1^{\Delta 44}$. Data are shown as the mean and SEM of 3 replicates.

Fig. S7.

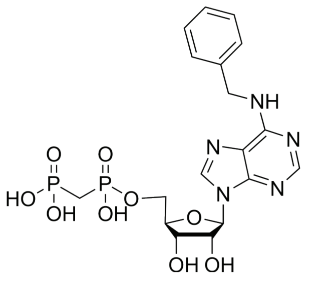

PSB12379

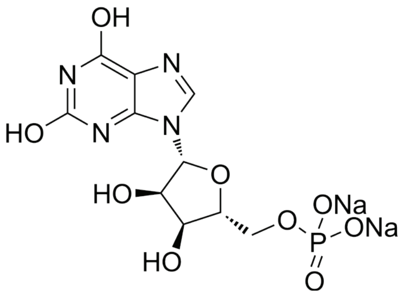

Xanthosine 5′
Monophosphate (Na salt)

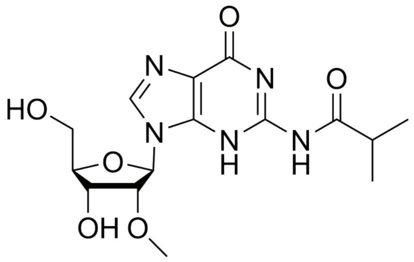

N2-Isobutyryl-2′-O-
methylguanosine

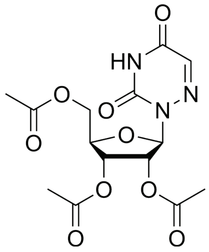

Azaribine

Fig. S7. **Chemical structures of selected nucleotide compounds.** The experiments using indicated compounds are featured in Figure 5 and Supplemental Figure S6.

Fig. S8.

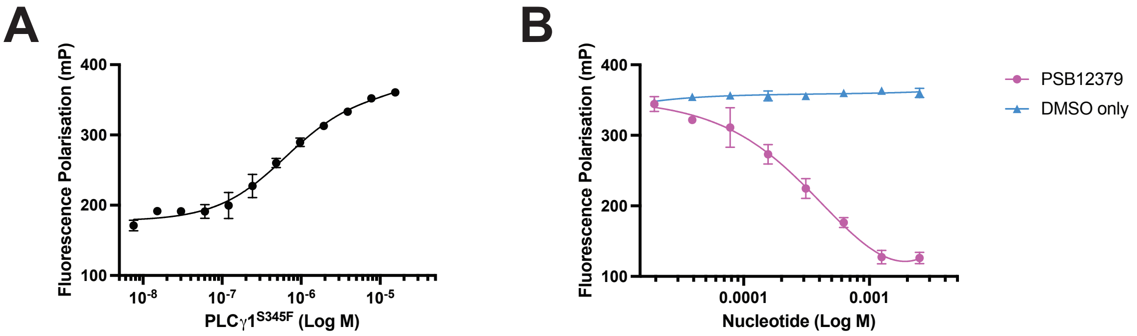

Fig. S8. Assessment of the binding of PSB12379 nucleotide compound to PLCγ1^{S345F} using fluorescence polarisation. (**A**)The concentration of PLCγ1^{S345F} is titrated against a fixed concentration of TNT-ATP (1 μM) and the fluorescence polarisation determined. (**B**) In a competition experiment, the highest concentration of PLCγ1^{S345F} is mixed with 1 μM TNT-ATP and titrated with increasing PSB12379. A parallel experiment with the PBS12379 solvent, DMSO, is also performed.

Table S1.

	Rat PLCγ1 S345F
**Data Collection**	
Beamline	I04 – Diamond Light Source
Wavelength (Å)	0.95374
Resolution Range (Å)	115.4 – 2.56 (2.60 – 2.56)*
Space group	$P\ 2_1\ 2_1\ 2_1$
Cell parameters a, b, c (Å)	72.44, 82.39, 230.68
Total reflections	547,294 (26,960)
Unique reflections	45,372 (2,240)
Multiplicity	12.1 (12.0)
Completeness (%)	100.0 (100.0)
Mean I/Sigma(I)	8.5 (1.4)
Wilson B-factor (Å^2)	68.0
R_{meas}	0.314 (4.381)
$CC_{1/2}$	0.987 (0.342)
**Refinement**	
Reflections used in refinement	45,193 (2,540)
Reflections used for R-free	2,289 (125)
Resolution Range (Å)	41.20 – 2.56 (2.62 – 2.56)
R_{work}/R_{free} (%)	18.81/23.33 (29.01/33.19)
Number of non-hydrogen atoms	9,399
Protein atoms	9,072
Solvent molecules	322
Ligand atoms	5
Protein residues	1116
B-factor (Å^2) - average	60.40
Protein	60.78
Solvent	49.43
Ligands	66.31
Ramachandran Plot	
Favoured (%)	96.11
Allowed (%)	3.81
Outliers (%)	0.00
Rotamer outliers (%)	1.21
All atom clash score	6.74
Rmsd	
Bonds (Å)	0.009
Angles (deg)	1.050
PDB code	9QB7

*Outer cell in parenthesis.

Table S1: Crystallographic data collection and refinement statistics
